# Direct observation of charge transfer between molecular heterojunctions based on inorganic semiconductor clusters†

**DOI:** 10.1039/d0sc00458h

**Published:** 2020-03-23

**Authors:** Chaozhuang Xue, Xing Fan, Jiaxu Zhang, Dandan Hu, Xiao-Li Wang, Xiang Wang, Rui Zhou, Haiping Lin, Youyong Li, Dong-Sheng Li, Xiao Wei, Daoyuan Zheng, Yang Yang, Keli Han, Tao Wu

**Affiliations:** College of Chemistry, Chemical Engineering and Materials Science, Soochow University Suzhou 215123 China wutao@suda.edu.cn; Jiangsu Key Laboratory for Carbon-Based Functional Materials & Devices, Institute of Functional Nano & Soft Materials (FUNSOM), Soochow University Suzhou Jiangsu 215123 China; Hubei Provincial Collaborative Innovation Center for New Energy Microgrid, Key Laboratory of Inorganic Nonmetallic Crystalline and Energy Conversion Materials, College of Materials and Chemical Engineering, China Three Gorges University Yichang 443002 China; School of Chemistry and Chemical Engineering, Shanghai Jiao Tong University Shanghai 200240 China; State Key Laboratory of Molecular Reaction Dynamics, Dalian Institute of Chemical Physics, Chinese Academy of Science Dalian 116023 China; Institute of Molecular Sciences and Engineering, Shandong University Qingdao 266235 China

## Abstract

A deep understanding of the dynamics of photogenerated charge carriers is extremely important for promoting their germination in semiconductors to enhance the efficiency of solar energy conversion. In contrast to that of organic molecular heterojunctions (which are widely employed in organic solar cells), the charge transfer dynamics of purely inorganic molecular heterojunctions remains unexplored. Herein, we reveal the dynamics of charge transfer between inorganic semiconductor molecular heteroclusters by selecting a group of open-framework metal chalcogenides as unique structure models constructed from supertetrahedral *T3-InS* ([In_10_S_20_]) and *T4-MInS* ([M_4_In_16_S_35_], M = Mn or Fe) clusters. The staggered band gap alignment in *T3-T4-MInS* molecular heterojunctions enables the photogenerated charge carriers to be directionally transferred from T3-InS clusters to adjacent T4-MInS clusters upon irradiation or application of an external electric field. The simultaneous independence of and interactions between such two heteroclusters are investigated by theoretical calculations, steady- and transient-state absorption/photoluminescence spectroscopy, and surface photovoltage analysis. Moreover, the dynamics of cluster-to-cluster-to-dopant photogenerated charge transfer is deliberately elucidated. Thus, this work demonstrates the direct observation of charge transfer between molecular heterojunctions based on purely inorganic semiconductor clusters and is expected to promote the development of cluster-based semiconductors for solar cells.

## Abstract 2

Charge transfer between inter-clusters is directly observed in inorganic molecular heterojunctions.

## Graphical abstract

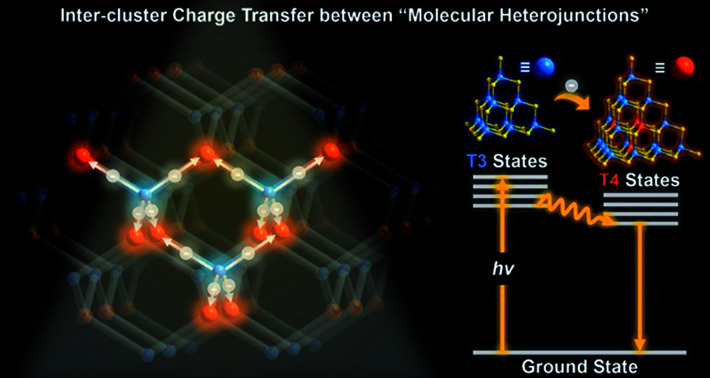

## Introduction

**Scheme 1:**
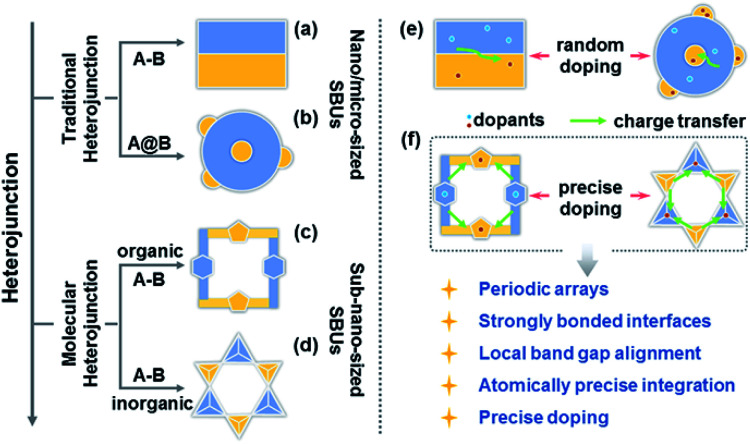
(a–d) Types of heterojunctions. (e and f) Comparison of charge transfer dynamics between traditional and molecular heterojunctions.

Charge transfer efficiency and direction are important kinetic factors predominantly impacting semiconductor performance in photoelectrochemical and photovoltaic applications.^1–4^ On the one hand, highly efficient transfer of photogenerated charge carriers enables the long-term separation of electron–hole pairs and improves the effectiveness of carrier utilization in enhancing photoelectric performance.^5–7^ On the other hand, exploration of explicit charge carrier migration pathways/directions can deepen our understanding of the complicated physical phenomena and catalytic reactions involving charge transfer processes.^1,8^ The construction of semiconductor heterojunctions with staggered band gap alignment is an effective strategy to promote the separation of photogenerated charge carriers and study charge migration dynamics.^9–12^ However, it remains challenging to achieve the desired charge separation efficiency and deeply understand the charge migration direction based on the information available for conventional semiconductor heterojunctions commonly synthesized by multistep procedures and comprising size-variable interfacial phases with a relatively unclear interface location, complicated interface structures, and randomly positioned dopants (Scheme 1a, b and e).

Over the past decade, crystalline molecular heterojunctions,^13–17^ especially those with strongly bonded interfaces, have drawn much attention because of their ideal contact areas, bonding sites between adjacent molecules (or phases), and controllable compositions in ultra-small subunits.^18–21^ This well-defined heterostructured configuration offers obvious advantages for photovoltaic and catalytic applications, as the sufficient hybridization of atomic orbitals permits efficient carrier delocalization and allows programmable band structures to meet the potential of catalytic reactions. The incorporation of secondary building units (SBUs) into crystalline skeletons through one-step self-assembly is an effective strategy to obtain molecular heterojunctions with periodic arrays, strongly bonded interfaces, staggered band gap alignment, and atomically precise integration (Scheme 1c and d), allowing one to assess the comprehensive impacts of molecular building blocks, energy levels, and dopants on the microscopic process of energy conversion. Interestingly, a crystalline molecular heterojunction based on covalent organic frameworks (COFs) has been recently reported (Scheme 1c), representing a diversified combination of organic SBUs with outstanding semiconduction and photoconduction properties.^18,22–26^ Moreover, the systematical assembly of donor and acceptor SBUs allows one to reveal some mechanistic details (*e.g.* exciton formation and charge separation/migration) for such ordered atomically precise molecular heterojunctions (Scheme 1f).^25,26^ Although the synthesis and charge dynamics of molecular COF heterojunctions have been extensively studied, the construction of purely inorganic semiconductor molecular heterojunctions and their photoelectric dynamic behaviours at the sub-nanoscale cluster size remain underexplored (Scheme 1d). Thus, the fabrication of inorganic molecular heterojunctions from different atomically precise SBUs with periodic arrays and controllable band structures is a task of high importance. Unfortunately, such model systems seem to be aspirational rather than feasible for current hot inorganic materials, such as insulating metal-oxide-based zeolites and polyoxometalates.

Crystalline inorganic metal chalcogenides with supertetrahedral clusters (denoted as T*n*, where *n* is the number of metal sites along the tetrahedron edge) serving as SBUs have been extensively investigated because of their fascinating architectures and the effective integration of porosity with semiconducting properties.^27–32^ Recently, discrete clusters with uniform size and atomically precise crystal lattice structure have been successfully dispersed into cluster-based quantum-dot-like nanoparticles (also called supraclusters) in solvents, and correlations between the cluster structure and function (such as electrochemical, photocatalytic and photoelectric applications) have been established.^33–37^ Although the roles of clusters in crystalline open frameworks remain unclear, the significance of clusters in these frameworks seems to extend beyond the beauty of a symmetrical structure and apparent functionality as nodes for open framework construction. For example, precise doping of clusters at the atomic scale allows one to study the synergistic effect of framework heteroatoms on photoluminescence and electrocatalytic properties.^31,34,38^ Moreover, the incorporation of multi-metal compositions with various stoichiometric ratios allows one to control the band structure of cluster-based materials for potential applications in photocatalytic pollutant degradation and photocatalytic fuel synthesis.^33,39^ From a structural perspective, two types of chalcogenide T*n* clusters can co-crystallize into 2D or even 3D frameworks with periodic A–B–A–B arrays.^40–44^ The compositions of cluster A and cluster B can be engineered independently to result in sub-nanoscale segregation of domains for possible charge separation. Such hybrid chalcogenide-cluster-based molecular heterojunctions with ordered charge-transporting 3D channels in a periodical lattice may provide an ideal platform for investigating the dynamics of photogenerated charge carriers (Scheme 1f).

**Fig. 1:**
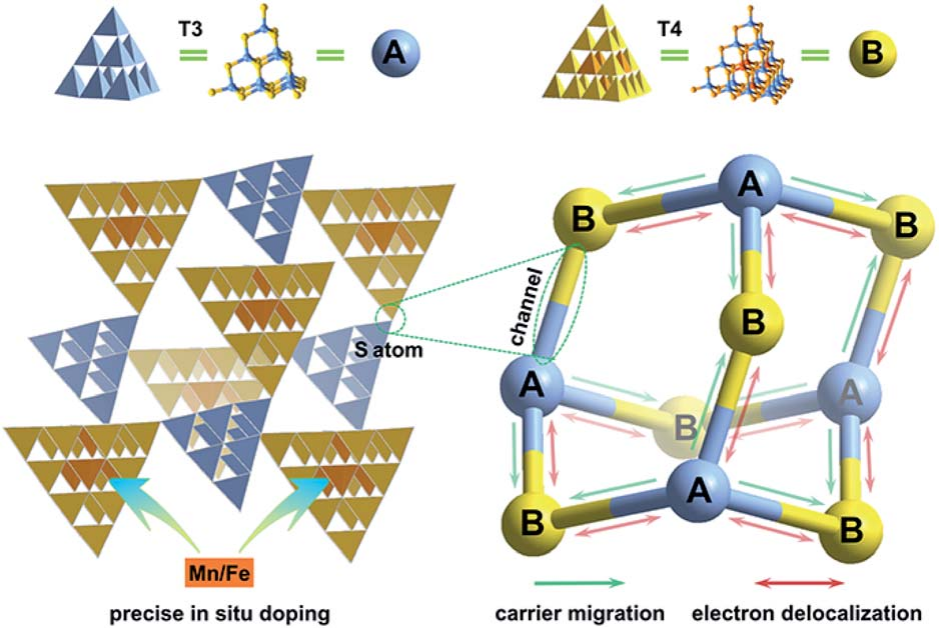
Schematics of charge transfer dynamics between sub-nanoscale hetero-clusters in 3D cluster-based open-framework chalcogenides (A = T3-InS, B = T4-MInS, M = Mn or Fe).

Herein, we take a significant step toward the direct observation of charge transfer between adjacent cluster-based domains in inorganic chalcogenide molecular heterojunctions (denoted as *T3-T4-MInS*, M = Mn or Fe), which feature sub-nanoscale supertetrahedral T3-InS and T4-MInS clusters alternately connected into an extended double-interpenetrated diamond-type framework corresponding to a sphalerite-type AB topology of two different tetrahedral clusters A and B that are connected by corner-sharing (Fig. 1). The results of theoretical calculations and UV-vis absorption spectroscopy indicate the staggered band structure of these molecular heterojunctions. In addition, the independence of and inter-cluster interactions between T3-InS and T4-MInS clusters as well as the dynamics of photogenerated charge carrier transfer between these clusters are discussed in detail from experimental and theoretical perspectives.

## Experimental section

### Chemicals and materials

Manganese acetate tetrahydrate (Mn(CH_3_COO)_2_·4H_2_O, 99.9%, powder), iron nitrate nonahydrate (Fe(NO_3_)_3_·9H_2_O, 99.9%, powder), indium (In, 99.9%, powder), sulfur (S, 99.9%, powder), 1,8-diazabicyclo[5.4.0]-7-undecene (DBU, >99%, liquid), 1,5-diazabicyclo[4.3.0]non-5-ene (DBN, 98%, liquid), *N*-aminoethylpiperazine (AEP, >99%, liquid), *N*,*N*-dimethylformamide (DMF, >99.8%, liquid) and deionized water were all used without further purification.

### Syntheses of *T3-T4-MInS*

Mn(CH_3_COO)_2_·4H_2_O powder (47 mg, 0.19 mmol), In powder (82 mg, 0.71 mmol), S powder (60 mg, 1.88 mmol), DBN (1 mL) and DMF (2 mL) were added into a 23 mL Teflon-lined stainless-steel autoclave and stirred for about 20 min. The reactors were then sealed and heated at 190 °C for 5 days. After washing with ethanol, bronzing octahedral crystals of *T3-T4-MnInS* were obtained (yield: 105 mg, ∼32% based on Mn). The synthetic method for T3-T4-FeInS is the same as that of *T3-T4-MnInS* except for substituting Mn(CH_3_COO)_2_·4H_2_O with 35 mg (0.09 mmol) of Fe(NO_3_)_3_·9H_2_O (yield: 150 mg, ∼95% based on Fe).

### Synthesis of *T3-InS*

In powder (103 mg, 0.90 mmol), S powder (154 mg, 4.81 mmol) and AEP (2 mL) were added into a 23 mL Teflon-lined stainless-steel autoclave and stirred for about 20 min. The reactors were then sealed and heated at 190 °C for 7 days. After washing with ethanol, pale yellow octahedral crystals of *T3-InS* were obtained (yield: 120 mg, ∼27% based on In).

### Synthesis of *T4-MInS*

Mn(CH_3_COO)_2_·4H_2_O powder (47 mg, 0.18 mmol), In powder (100 mg, 0.87 mmol), S powder (120 mg, 3.75 mmol), DBU (1.5 mL), AEP (3 mL) and H_2_O (1 mL) were added into a 23 mL Teflon-lined stainless-steel autoclave and stirred for about 20 min. The reactors were then sealed and heated at 190 °C for 5 days. After washing with ethanol, pale yellow crystals *T4-MnInS* were obtained (yield: 90 mg, ∼40% based on Mn). Synthetic methods of *T4-FeInS* are the same as that of *T4-MnInS* except for substituting Mn(CH_3_COO)_2_·4H_2_O with 30 mg (0.06 mmol) Fe(NO_3_)_3_·9H_2_O (yield: 75 mg, ∼87% based on Fe).

### Structural characterization

Single-crystal X-ray diffraction measurements were performed on a Bruker Photon II CPAD diffractometer with graphite monochromated Mo Kα (*λ* = 0.71073 Å) radiation at 223 K. The structure was solved by the direct method using SHELXS-2014 and the refinement against all reflections of the compound was performed using SHELXL-2014. The protonated organic amines located in the extra framework cannot be identified due to their serious disorder and hence the squeeze subprogram has been performed. PXRD data of all compounds were collected on a desktop diffractometer (D2 PHASER, Bruker, Germany) using Cu-Kα (*λ* = 1.54056 Å) radiation operated at 30 kV and 10 mA.

### Theoretical calculations

All the theoretical calculations in this work were performed using Vienna *ab initio* simulation packages (VASP) with the projector-augmented wave (PAW) method. The Perdew–Burke–Ernzerhof (PBE) functional is used for the exchange and correlation interaction between electrons. The plane wave cutoff energy for this system was set to 500 eV. The Brillouin zone was sampled by 1 × 1 × 1 Monkhorst–Pack *k*-point sampling for structural optimization and self-consistent calculations. The internal coordinates of each system are fully optimized until the residual Hellmann–Feynman forces are smaller than 0.02 eV Å^−1^. The criterion of convergence of energy is set to 1 × 10^−4^ eV. The variations in vdW contributions of atoms in a local chemical environment were evaluated by the method of Tkatchenko and Scheffler (DFT-TS). Three methods have been tried to deal with the negative charges carried by the clusters. In the first approach, hydrogen atoms were added to the sulfur atoms to balance the negative charges. However, the added H atoms resulted in significant structural distortions upon structural relaxations. In the second approach, background positive charges were added to the unit cells by tuning the values of “NELECT” in the INCAR file. Nevertheless, the convergence of electronic structures becomes very poor, indicating that there are too many background charges and the obtained electronic states become unreliable. In the third approach, we did not try to balance the negative charges in calculations. The reason is that under realistic conditions, the negative charges are localized on the S atoms, while the positive charges of ligands are localized somewhere near the clusters. Thus, the experimentally measured properties are actually based on the electronic structures of clusters which carry some negative charges. We therefore select the third treatment in the present work.

### UV-vis absorption

Room-temperature solid-state UV-vis diffusion reflectance spectra of all the powder samples were measured on a Shimadzu UV-3600 UV-vis-NIR spectrophotometer by using BaSO_4_ as the reflectance reference. The absorption spectra were obtained from reflectance spectra by using the Kubelka–Munk function: *F*(*R*) = *α*/*S* = (1 − *R*)^2^/2*R*, where *R*, *α*, and *S* are the reflection, the absorption and the scattering coefficient, respectively.

### Photoluminescence (PL) characterization

PL and PLE measurements were measured using a HORIBA Scientific Fluorolog-3 steady state and time-resolved fluorescence spectrophotometer equipped with a 450 W xenon lamp. PL decays were measured by using a HORIBA Scientific Fluorolog-3 steady state fluorimeter with a time-correlated single-photon counting (TCSPC) spectrometer and a pulsed xenon lamp as the excitation source. Low temperature PL spectra were also recorded on a HORIBA Scientific Fluorolog-3 spectrophotometer with low temperature equipment.

### Surface photovoltage (SPV) measurements

Surface photovoltage (SPV) measurements were carried out using a system that includes a lock-in amplifier (SR830-DSP) with a light chopper (SR540), a source of monochromatic light, a photovoltaic cell, and a computer. Monochromatic light was provided by a 500 W xenon lamp (CHFXQ500 W, Global Xenon Lamp Power) and a double-prism monochromator (Zolix SBP500). The contact between samples and the electrode of fluorine-doped tin oxide (FTO) was resistance-free in measurements of SPV. The configuration of the photovoltaic cell is a sandwich-like structure of FTO–sample–FTO.

### Ultrafast transient absorption (TA) and emission measurements

Nanosecond transient absorption and emission measurements were carried out on powder samples by using laser flash photolysis (LFP) with a pump–probe system. The 355 nm pump pulse with a pulse width of 6 ns and energy of 30 mJ was generated by a Nd-YAG nanosecond laser system operated at a repetition rate of 3 Hz. A pulsed 450 W xenon lamp served as the probe light which scanned over the wavelength range of 360–700 nm. After a high intensity monochromator (WDG30-Z, Beijing Optical Instrument Factory), the signal was led to a photomultiplier tube (PMT, R928) for detecting transient absorption curves and emission decay curves.

## Results and discussion

### Construction of T3-T4-MInS molecular heterojunctions

**Fig. 2:**
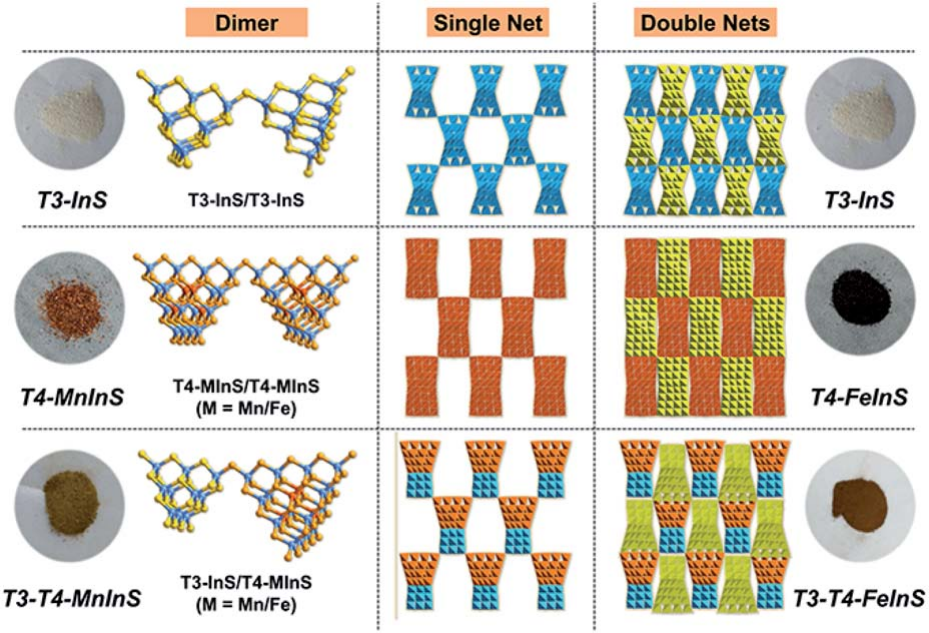
Images of *T3-InS*, *T4-FeInS*, *T4-MnInS*, *T3-T4-FeInS* and *T3-T4-MnInS*, as well as the corresponding structures.

Crystalline *T3-T4-MInS* samples, constructed from supertetrahedral T3-InS and T4-MInS clusters, were obtained by facile one-step solvothermal synthesis (see ESI† for details; note that italicized T*n* represents the cluster-based material, while non-italicized T*n* stands for the cluster itself). To directly reflect the dynamics of charge transfer/migration in *T3-T4-MInS* molecular heterojunctions, Mn^2+^ and Fe^2+^ ions were selected to control the electronic structure of T4-MInS clusters. Crystalline *T3-T4-MnInS* and *T3-T4-FeInS* appeared yellow and chocolate-colored, respectively (Fig. 2). Single-crystal X-ray structural analysis revealed that tetrahedrally connected T3-InS and T4-MInS clusters were alternately linked to each other through corner-shared S atoms to afford a 3D framework with a double-interpenetrated diamond topology, being isostructural and isomorphous with UCR-19 (Fig. S1–S3†).^42^ Compared to T3-InS and T4-MInS clusters in previously reported UCR-7 and UCR-5 compounds,^45^ respectively, T3-InS and T4-MInS clusters in *T3-T4-MInS* retained their pristine atomic arrangement, *i.e.*, each T3-InS cluster contained In^3+^ and S^2−^, and each T4-MInS cluster comprised In^3+^, S^2−^ and M^2+^ ions, with low-valent M^2+^ cations being precisely located on super-tetrahedron faces to meet Pauling's electrostatic valence rule. Theoretically, the physicochemical properties of T*n* clusters (including band structure and electron energy levels) depend on their compositions. Thus, the incorporation of chalcogenide heteroclusters with different compositions into the superlattice may create molecular heterojunctions with periodic arrays of domains and staggered band structure.

### Theoretical calculations

**Fig. 3:**
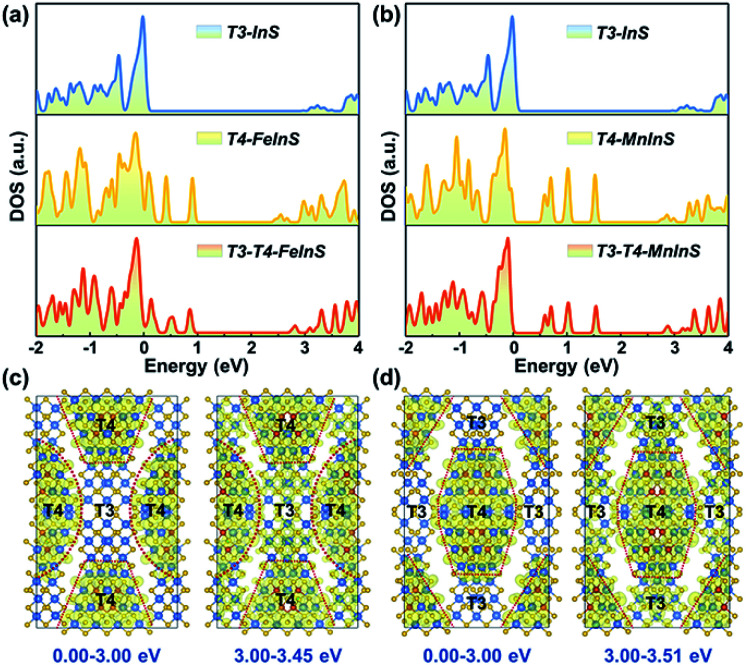
(a) DOS of *T3-InS*, *T4-FeInS*, and *T3-T4-FeInS*. (b) DOS of *T3-InS*, *T4-MnInS*, and *T3-T4-MnInS*. Band-decomposed charge densities in energy ranges of 0.00–3.00 eV and 3.00–3.45 eV for (c) *T3-T4-FeInS* and (d) *T3-T4-MnInS*. The isosurface value is 0.001 eV Å^−3^. S, In, Fe, and Mn atoms are presented with yellow, brown, blue, and violet circles, respectively.

To identify the spatial localization or distribution of the electronic bands of *T3-T4-MInS* for different molecular clusters and the strong charge interaction between them, theoretical calculations were performed on *T3-T4-MInS* and its contrast structures of pure *T3-InS* (UCR-7) and *T4-MInS* (UCR-5) using density functional theory (DFT) (Fig. 3). As shown in Fig. 3a and b, the densities of states (DOS) of *T3-T4-MInS* with characteristic semiconducting features seemed to be a simple combination of the DOS of *T3-InS* and *T4-MInS*, which suggested that the partial band structures of both T3-InS and T4-MInS were retained in *T3-T4-MInS*. To probe charge distribution in T3-InS and T4-MInS clusters, DOS peaks corresponding to excited states were analyzed by meticulous peak separation and resolution. For *T3-T4-FeInS*, the excited states in the energy window of 3.00–3.45 eV were localized at both T3-InS and T4-FeInS clusters, and when the selected energy range was shifted to <3.00 eV, the electronic states were only seen in T4-FeInS clusters (Fig. 3c). Similarly, the excited states of *T3-T4-MnInS* in the energy range of 3.00–3.51 eV were distributed in both T3-InS and T4-MnInS clusters, while those below 3.00 eV were only present in T4-MnInS clusters (Fig. 3d). In hybrid clusters, T4-MInS and T3-InS units were connected through corner-shared S linkers (also denoted as the interface), as highlighted in red in Fig. S4a and b.† The projected DOS (PDOS) (Fig. S4c and d†) and band-decomposed charge densities (Fig. 3c and d) show that these linker S atoms possessed the exited states of both T4-MInS and T3-InS clusters, suggesting that charge transfer between T3-InS and T4-MInS clusters was enabled by the presence of corner-shared S atoms acting as gangplanks. Correspondingly, hot electrons injected into empty states located 3.00 eV above the Fermi level were expected to be distributed in both T3-InS and T4-MInS clusters. However, upon electronic relaxations, electrons in T3-InS may be transferred to lower energy levels (empty states between 0.00 and 3.00 eV) of T4-MInS *via* linker S atoms.

### Absorption spectra and optical band gap

**Fig. 4:**
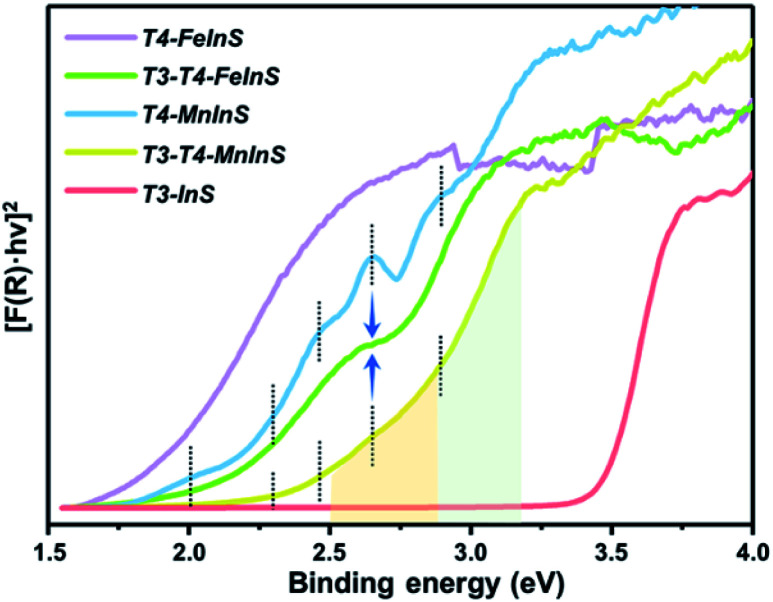
Transformed Kubelka–Munk spectra of *T3-InS*, *T4-MInS* and *T3-T4-MInS*.

To verify theoretical results, solid-state UV-vis absorption measurements were carried out on as-synthesized crystalline *T3-T4-MInS* (Fig. 4 and S5†) as well as on *T3-InS* and *T4-MInS* for comparison (Fig. S1 and S6†). Fig. 4 shows the transformed Kubelka–Munk spectra of *T3-InS*, *T4-MInS* and *T3-T4-MInS*. As can be seen, band gaps of *T3-InS*, *T4-MnInS* and *T4-FeInS* were determined to be equal to 3.4, 2.3, and 1.8 eV, respectively. These results strongly suggest that divalent metal ions play an important role in controlling the band structure of cluster-based chalcogenides. Interestingly, heterocluster-based *T3-T4-MnInS* exhibited abnormal Tauc plots, which, in the ranges of 2.5–2.9 and 2.9–3.2 eV, featured double slopes assumed to originate from T4-MnInS and T3-InS & T4-MnInS clusters, respectively. Electron delocalization between T3-InS and T4-MnInS clusters led to a roughly compromised band gap in *T3-T4-MnInS*, as compared to pure *T3-InS* and pure *T4-MnInS*. This phenomenon was more obvious for *T3-T4-FeInS*, in which case a plateau at 2.6–2.7 eV observed between a double band edge suggested the existence of band alignment and the relative independence of T3-InS and T4-FeInS clusters in the band structure. In addition, *T4-MnInS* and *T3-T4-MnInS* displayed five obvious absorption peaks at 2.0, 2.3, 2.4, 2.6, and 2.9 eV, attributed to the characteristic d–d transitions of Mn^2+^ ions from the ground state [^6^A_1_(^6^S)] to excited states [^4^E(^4^D), ^4^T_2_(^4^D), ^4^E(^4^G) or ^4^A_1_(^4^G), ^4^T_2_(^4^G), and ^4^T_1_(^4^G)].^46^ No characteristic transitions of Fe^2+^ were observed in the Tauc plot of *T4-FeInS*. These results suggest that compared to Fe^2+^ ions in the T4-FeInS cluster, Mn^2+^ ions in the T4-MnInS cluster feature a lower hybridization degree, in agreement with DFT results (Fig. S7†).

### Photoluminescence (PL) properties of T3-T4-MnInS

**Fig. 5:**
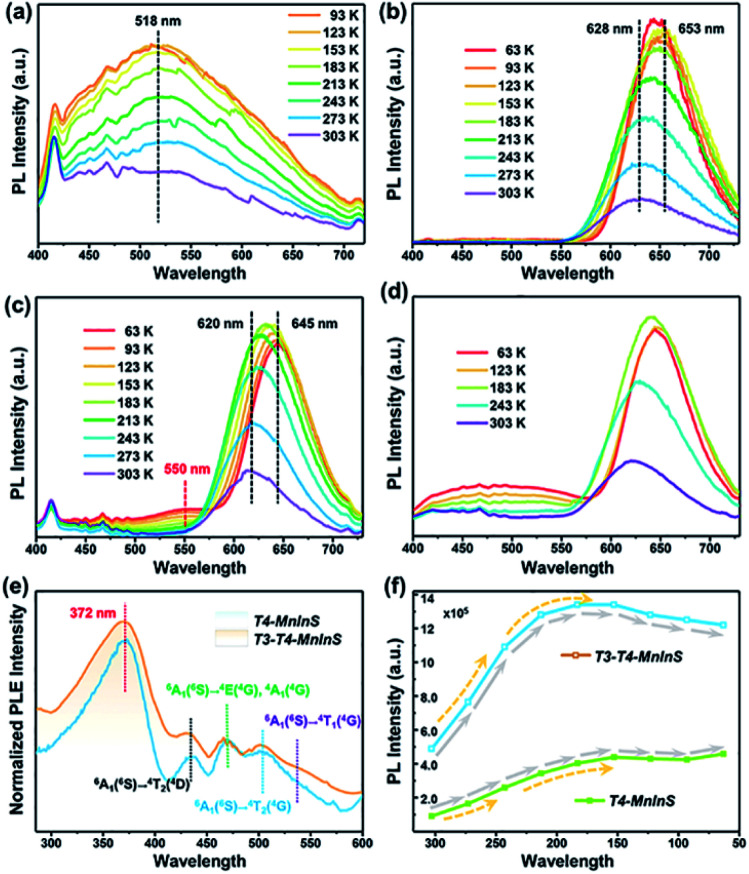
PL spectra of (a) *T3-InS*, (b) *T4-MnInS*, (c) *T3-T4-MnInS* and (d) a mechanical mixture of *T3-InS* and *T4-MnInS* (1 : 1, mol mol^−1^) upon excitation at 375 nm and different temperatures. (e) Normalized PLE spectra of *T4-MnInS* and *T3-T4-MnInS* for emission at 631 nm at room temperature. (f) Temperature-dependent PL intensity of *T4-MnInS* and *T3-T4-MnInS*.

The simultaneous independence of and inter-cluster interactions between T3-InS and T4-MnInS clusters in the molecular heterojunctions of *T3-T4-MnInS* were further revealed by PL spectroscopy. The incorporation of Mn^2+^ into chalcogenide T*n* clusters resulted in Mn^2+^-related PL emission based on indirect excitation *via* exciton charge (or energy) transfer from the host cluster lattice to Mn^2+^ and/or the direct excitation of Mn^2+^.^38,47^ To directly observe the photogenerated charge transfer dynamics between T3-InS and T4-MnInS clusters, temperature-dependent PL measurements were carried out on *T3-InS*, *T4-MnInS*, and *T3-T4-MnInS* polycrystals. As shown in Fig. 5a and S8,†*T3-InS* exhibited weak PL emission at room temperature (RT) upon excitation at 350, 375, and 400 nm, and the full width at half maximum of PL emission (zenith at 518 nm) increased with decreasing temperature. In contrast, high-intensity RT PL emission was observed for *T4-MnInS* and *T3-T4-MnInS* excited under the same conditions as *T3-InS* (Fig. 5b, c and S9†). For *T4-MnInS*, RT PL emission with a maximum at ∼628 nm was attributed to a split d-orbital transition (^4^T_1_ → ^6^A_1_) in the 3d^5^ shell of Mn^2+^, and the possible band-edge emission was suppressed because of charge (or energy) transfer from the host cluster to Mn^2+^ inside of the cluster.^38,48^ To better understand the independence of and inter-cluster interactions between T3-InS and T4-MnInS clusters in such molecular heterojunctions, we classified the above PL emission into T3-InS emission (denoted as T3-em) and T4-MnInS emission (denoted as T4-em). Temperature-dependent PL emission spectra of *T3-T4-MnInS* showed single strong Mn^2+^-related emission with a maximum at ∼620 nm at RT, while another wide emission band at 475–575 nm (zenith at 550 nm) emerged and gained intensity with decreasing temperature (Fig. 5c), in stark contrast to the behavior of *T4-MnInS* (Fig. 5b) and the mechanical mixture of pure *T3-InS* and *T4-MnInS* (Fig. 5d). Compared to that of *T4-MnInS*, the additional blue PL emission of *T3-T4-MnInS* was ascribed to T3-em in view of the structural difference between *T4-MnInS* and *T3-T4-MnInS*, in which half of the T4-MnInS clusters in *T4-MnInS* are substituted by T3-InS clusters. The co-existence of T3-em and T4-em in the case of *T3-T4-MnInS* further demonstrated the relatively separated electronic structures of T3-InS and T4-MnInS clusters, which possibly endowed them with relatively independent photoelectric properties. Even so, the delocalization of electrons between T3-InS and T4-MnInS clusters could still be realized through bridging S atoms, as suggested by the variation of T3-em. The T3-em of *T3-InS* at ∼518 nm was dramatically red shifted to 550 nm in *T3-T4-MnInS*, which was ascribed to the delocalization of electrons between heteroclusters. Therefore, the PL performance of *T3-T4-MnInS* was concluded to be a concretization of the calculated results on both the independence of and inter-cluster interactions between T3-InS and T4-MnInS clusters.

The oriented transfer of photogenerated charges from T3-InS clusters to T4-MInS clusters is a fascinating dynamic feature. To further probe charge transfer between heteroclusters, we performed PL excitation (PLE) measurements for *T4-MnInS* and *T3-T4-MnInS* (Fig. 5e). The PLE spectra of *T4-MnInS* and *T3-T4-MnInS* featured five wide excitation peaks at 372, 433, 469, 502, and 535 nm. Peaks 2–5 were assigned to transitions from the ^6^A_1_(^6^S) ground state to ^4^T_1_(^4^G), ^4^T_2_(^4^G), ^4^E(^4^G) or ^4^A_1_(^4^G), and ^4^T_2_(^4^D) states. This finding was consistent with peaks in the corresponding absorption spectra and suggested that Mn^2+^ ions in both *T4-MnInS* and *T3-T4-MnInS* can be directly excited. It is worth noting that for *T4-MnInS*, the strongest PLE peak at ∼372 nm was attributed to charge transfer from the host cluster to Mn^2+^ ions, as these ions in the T4-MnInS cluster cannot be directly and effectively excited at this wavelength.^46^ It should be noted that the typical absorption peak of Mn^2+^ ions in *T4-MnInS* that is involved with the transition from ^6^A_1_(^6^S) to ^4^E(^4^D) cannot be observed in its room-temperature PLE spectrum. This is possibly because Mn^2+^ ions in the crystal lattice possess a relatively low d–d transition possibility or absorption coefficient at room temperature, and the weak PLE peak in the range of 375–410 nm is overlapped by the strong and wide PLE peak in a wide range of 350–410 nm. This assumption was further supported by low-temperature PLE spectra, in which the transition from ^6^A_1_(^6^S) to ^4^E(^4^D) was clearly observed (Fig. S10a†). In addition, charge transfer within heteroclusters was further confirmed by variation of temperature-dependent PL emission intensity. It is well known that Mn^2+^-related PL emission intensity increases with decreasing because of the concomitant suppression of non-radiative transitions; however, the carrier transfer rate in such semiconductors is theoretically expected to decrease with decreasing testing temperature.^49^ As expected, the degree of PL intensity enhancement of *T4-MnInS* and *T3-T4-MnInS* upon excitation at 375 nm increased when the temperature decreased from RT to 243 K (Fig. 5f), which suggested that the suppression of non-radiative transition dominated over the recombination dynamics of carriers. However, the degree of PL intensity enhancement decreased at temperatures below 243 K. Given that the excitation wavelength of 375 nm dominates the charge-transfer-induced Mn^2+^-related PL emission, the above slowdown in the degree of PL intensity enhancement was ascribed to the suppression of host-to Mn^2+^ carrier transfer at low temperature. In particular, *T3-T4-MnInS* showed strong PL emission intensity that was maximized at 183 K, *i.e.*, at an equilibrium state of two kinetic controlling factors. However, for *T4-MnInS*, this equilibrium state was obtained at a much lower temperature of 153 K. The temperature-dependent parabolic PL intensity curve also suggested that although temperature strongly affects charge transfer dynamics, T3-InS clusters significantly contribute to Mn^2+^-related PL emission in such molecular heterojunctions constructed from T3-InS and T4-MnInS clusters.

### PL decay dynamics and electron paramagnetic response signals

**Fig. 6:**
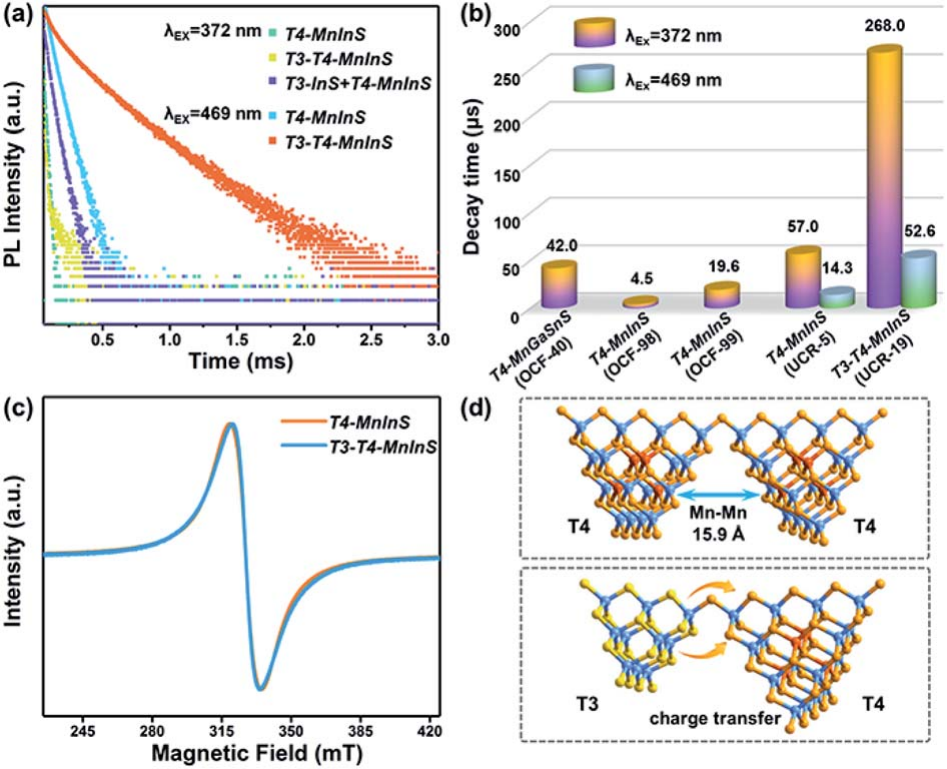
(a) PL decay curves of *T3-T4-MnInS*, *T4-MnInS*, and the mechanical mixture of *T3-InS* and *T4-MnInS* obtained for pulsed 372 nm (or 469 nm) excitation and 631 nm emission at RT. (b) Average PL decay lifetimes of *T3-T4-MnInS* and other T4-MnInS-cluster-based chalcogenides reported previously. (c) EPR spectra of *T4-MnInS* and *T3-T4-MnInS*. (d) Schematic structural difference between T4–T4 and T4–T3.

To further evaluate the impact of the T3-InS cluster on T4-em from the T4-MnInS cluster, we probed time-resolved PL dynamics for *T4-MnInS* and *T3-T4-MnInS* using time-correlated single photon-counting (TCSPC) experiments and fitted the obtained curves by a multi-exponential function: *I*(*t*) = ∑*A*_i_ exp(−*t*/*τ*_i_) (Table S1†), with average lifetimes determined as *τ*_ave_ = ∑*A*_i_*τ*_i_^2^/∑*A*_i_*τ*_i_. Fig. 6a presents the RT PL decay dynamics of emission at 631 nm for both samples excited at 372 nm. The decay curves were fitted by a tri-exponential method, and the long-term average decay time of *T3-T4-MnInS* was calculated as 268 μs, being almost five-fold longer than that of *T4-MnInS* (57 μs) and ten-fold longer than that of other T4-MnInS-based 0D and 2D materials reported previously (Fig. 6b).^46,50^ These results indirectly suggest the existence of exciton (or charge) transfer from the T3-InS cluster to the T4-MnInS cluster, which prolongs the lifetimes of Mn^2+^-related PL. Moreover, the occurrence of the above charge transfer was further indirectly verified by the PL decay dynamics of *T4-MnInS* and *T3-T4-MnInS* excited at 469 and 504 nm. During this excitation, Mn^2+^ ions in T4-MnInS clusters were excited directly, and the possible inter-cluster charge transfer was therefore completely ruled out. The PL decay lifetimes of both *T4-MnInS* and *T3-T4-MnInS* excited at 469 and 504 nm were much shorter than those obtained upon excitation at 372 nm (Fig. 6a, b and S11†). The PL lifetime of *T3-T4-MnInS* excited at 469 nm was only 20% that obtained for excitation at 372 nm, which demonstrates the importance of adjacent T3-InS clusters in *T3-T4-MnInS* as charge donors for increasing the lifetime of T4-em decay. In addition, it should be noted that distance-directed Mn–Mn interactions (dipolar–dipolar interactions) can also affect the PL lifetime.^51^ In *T4-MnInS* and *T3-T4-MnInS*, Mn–Mn interactions between Mn^2+^ ions in adjacent clusters can be ignored because of the large Mn⋯Mn distance (>1.5 nm) (Fig. 6d). In addition, there is also no difference between the local coordination configuration of Mn^2+^ ions in *T4-MnInS* and *T3-T4-MnInS* because of the identical distribution of these ions in the T4-MnInS cluster, as confirmed by EPR experiments. Fig. 6c shows that the EPR spectra of both *T3-T4-MnInS* and *T4-MnInS* featured centrosymmetric broad peaks of identical width. Therefore, charge transfer from T3-InS to T4-MnInS clusters was concluded to be the main reason of the long decay lifetime of *T3-T4-MnInS*.

### PL properties of T3-T4-FeInS

**Fig. 7:**
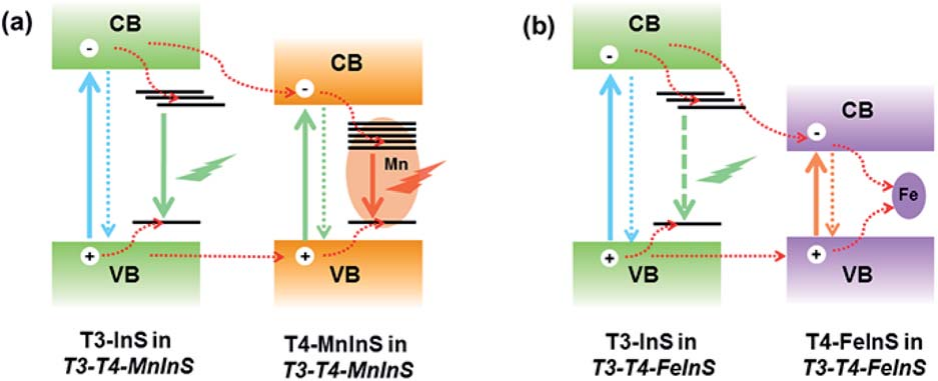
Dynamics of photogenerated charge carrier transfer in (a) *T3-T4-MnInS* and (b) *T3-T4-FeInS*.

In *T3-T4-FeInS*, charge transfer between heteroclusters was more distinct because of the complete quenching of T3-em PL. PL measurements of *T4-FeInS* and *T3-T4-FeInS* for excitation at 350, 375, and 400 nm at RT and low temperature (Fig. S12 and S13†) failed to detect PL emission signals, which was attributed to the incorporation of Fe^2+^ ions acting as good non-radiative recombination centers of electron–hole pairs.^52,53^ Given that T3-InS and T4-FeInS clusters in *T3-T4-FeInS* were confirmed to be independent units by theoretical calculations and UV-vis absorption spectroscopy, the orbital hybridization of Fe^2+^ ions with S^2−^ anions was thought to be confined to the T4-FeInS cluster. Therefore, we inferred that the suppression of possible T3-em PL may be ascribed to the photogenerated charge carrier transfer from T3-InS clusters to T4-FeInS clusters, identical to the dynamic process occurring in *T3-T4-MnInS* except for the different combination channels, *i.e.*, non-radiative transition within Fe^2+^ ions in the former case and radiative transition within Mn^2+^ ions in the latter case (Fig. 7).

### Surface photovoltage (SPV) measurements

**Fig. 8:**
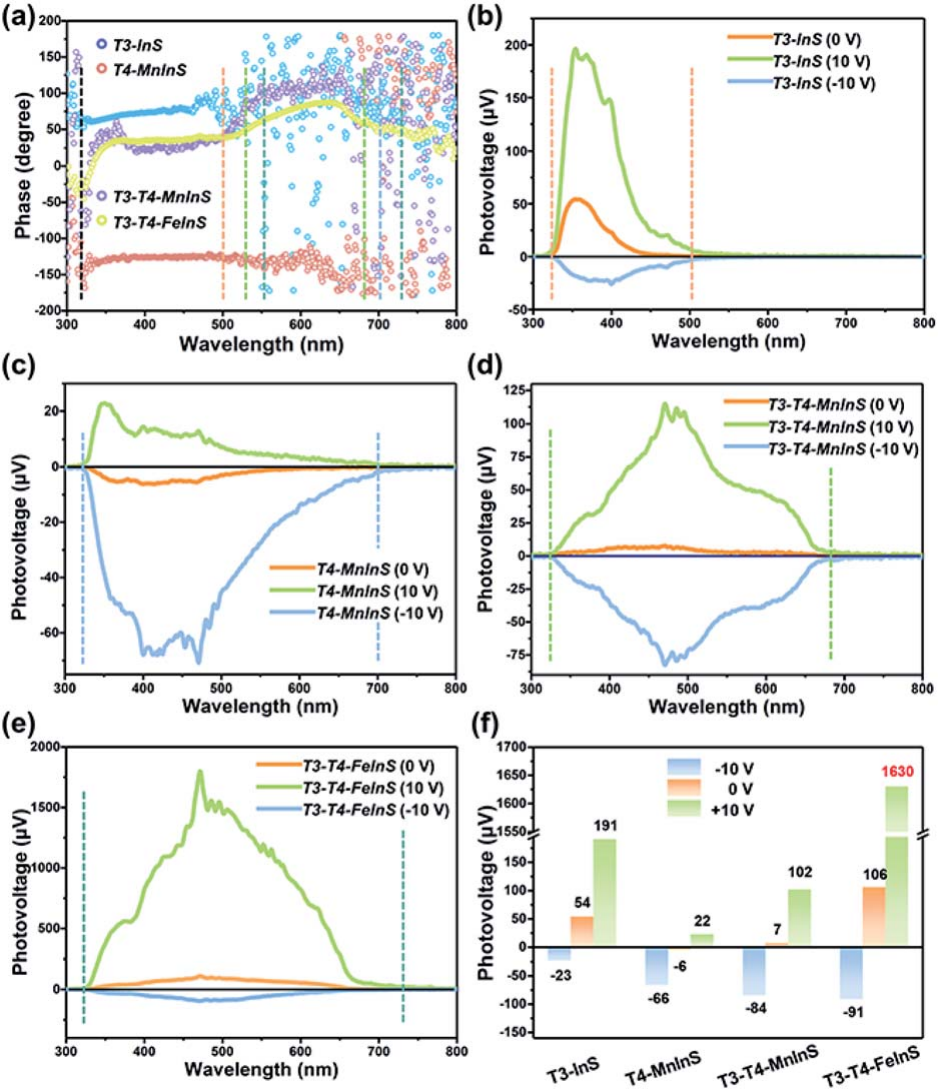
(a) SPV phase spectra of *T3-InS*, *T4-MnInS*, *T4-FeInS*, *T3-T4-MnInS* and *T3-T4-FeInS* without applied bias. (b–f) SPV spectra of (b) *T3-InS*, (c) *T4-MnInS*, (d) *T3-T4-MnInS*, (e) *T4-FeInS* and (f) *T3-T4-FeInS* recorded at biases of 0, 10, and −10 V.

SPV measurements were used to investigate the performance of the photogenerated charge carriers in *T3-T4-MInS* molecular heterojunctions. Fig. 8 shows the SPV phase spectra of *T3-InS*, *T4-MnInS* and *T3-T4-MInS* powder crystals for front-side illumination (*i.e.* samples were first directly irradiated by monochromatic light from the top electrode side) without applied bias and the corresponding SPV spectra measured at biases of 0, 10, and −10 V. The spectra of *T3-InS* and *T4-MnInS* featured approximately straight-line phase curves, indicating the occurrence of single-phase photoresponses throughout the SPV response region. The phase values of *T3-InS* and *T4-MnInS* equaled 70° and −150°, respectively (Fig. 8a), implying that photogenerated holes were generally transported to the top electrode for *T3-InS*, while photogenerated electrons were transported to the top electrode for *T4-MnInS* because of the intrinsic band potential difference between the electrode and samples attached on the electrode surface (denoted as the Schottky potential barrier).^54,55^ The fact that charge carrier transfer in *T4-MnInS* occurs in a direction opposite to that observed in *T3-InS* suggests intrinsic differences in semiconducting nature of these two species. In contrast, no SPV response for *T4-FeInS* was observed even when bias was applied (Fig. S14†). This finding was ascribed to the intrinsic electronic properties of T4-FeInS-based chalcogenide materials, as a similar sluggish SPV response of T4-FeInS was also observed for crystalline UCR-1-FeInS, which is also constructed from T4-FeInS clusters. The rather weak SPV response of UCR-1-FeInS was even independent of the external electric field, with the phase value being approximately zero (Fig. S15†). For hybrid molecular heterojunctions between T3-InS and T4-MInS, the phase values of both *T3-T4-MnInS* and *T3-T4-FeInS* were positive throughout the SPV response region. However, one could still notice the SPV response difference in phase value variation between T3-InS and T4-MInS clusters. As shown in Fig. 8a, the SPV phase curves of *T3-T4-MInS* could be divided into two response bands (in reference to UV-vis absorption spectra), *i.e.*, 320–520 and 520–680 nm for *T3-T4-MnInS*, and 320–550 and 550–730 nm for *T3-T4-FeInS*. The former band of *T3-T4-MInS* was attributed to the synergism of T3-InS and T4-MInS clusters, and the latter band was attributed to the T4-MInS cluster on account of light absorption ability. In addition, we noticed that the T3-InS-related SPV response (320–520 nm) of *T3-T4-MnInS* was dramatically red-shifted compared to that of *T3-InS* (320–500 nm); however, the cut-off wavelength of the SPV response corresponding to the T4-MnInS cluster (∼680 nm) was blue-shifted compared to that of *T4-MnInS* (∼700 nm). The extended SPV response of the T3-InS cluster to long wavelengths was also observed for *T3-T4-FeInS*. It is worth noting that the T4-FeInS cluster in *T3-T4-FeInS* exhibited an obvious SPV response at 550–730 nm, which was very different from the behavior of *T4-FeInS* and was ascribed to the electronic structure remodeling originating from the delocalization of electrons in T3-InS and T4-FeInS clusters.

Highly efficient charge transfer between adjacent heteroclusters was also manifested through SPV performance. When bias was applied during measurements to promote the separation and migration of photogenerated charge carriers, all samples exhibited positive phase values at a bias of 10 V bias and negative phase values at a bias of −10 V bias, which suggested that photogenerated holes accumulated on the top electrode under a strong positive electric field, while photogenerated electrons accumulated on the top electrode under a strong negative electric field (Fig. S16†). One can see that *T3-InS* exhibited a good positive photovoltage response (∼54 μV) in the absence of applied bias despite suffering from the instantaneous recombination of photogenerated carriers, as suggested by the corresponding photoelectric response (Fig. S17†). The positive SPV signal intensity sharply increased to 191 μV when a bias of 10 V was applied, while only a small negative SPV signal of about −23 μV was observed under a bias of −10 V (Fig. 8b and f). Inversely, *T4-MnInS* exhibited a weak negative signal of about −6 μV in the absence of applied bias, an increased negative SPV response of −66 μV at a bias of −10 V, and a small positive SPV response of about 22 μV at a bias of 10 V bias (Fig. 8c and f). The above difference between *T3-InS* and *T4-MnInS* was attributed to that in the intrinsic semiconducting nature of T3-InS and T4-MnInS clusters. Although the strong external electric field could control positive or negative charge accumulation at the top electrode, the SPV response intensity could be tuned and influenced by the type of Schottky potential barrier. Interestingly, the difference between the Schottky potential barriers of the T3-InS (and/or T4-MnInS) cluster and the top electrode was still observed for *T3-T4-MnInS* and impacted the SPV performance. As shown in Fig. 8d and f, under a zero external electric field, *T3-T4-MnInS* exhibited a weak positive SPV signal of 7 μV, which was opposite to that observed for *T4-MnInS* and much smaller than that observed for *T3-InS*. This behavior was ascribed to the local opposite surface electric fields in T3-InS and T4-MnInS cluster regions. Surface T3-InS clusters in *T3-T4-MnInS* dominated the transport of positively charged holes into the electrode under positive bias, whereas surface T4-MnInS clusters in *T3-T4-MnInS* dominated electron migration into the electrode under negative bias, which resulted in SPV responses of ∼102 and −84 μV under positive and negative electric fields, respectively. Notably, the negative SPV peak of *T3-T4-MnInS* was more intense than that (−66 μV) of *T4-MnInS*, although in *T3-T4-MnInS*, the amount of surface T4-MnInS clusters attached on the top electrode was less than that in *T4-MnInS*, as half of these clusters were replaced by sluggish T3-InS clusters at the electrode surface (the T3 : T4 cluster molar ratio of *T3-T4-MnInS* is 1 : 1). The enhanced SPV response was attributed to the more favorable charge transfer from T3-InS clusters to T4-MnInS clusters in *T3-T4-MnInS* on account of the existence of additional band alignment in *T3-T4-MnInS*. This SPV enhancement induced by band alignment was carried forward in the SPV performance of *T3-T4-FeInS* under a positive electric field. As shown in Fig. 8e and f, at a bias of 10 V, *T3-T4-FeInS* exhibited a very strong SPV response of ∼1630 μV, which was eight-fold higher than that of *T3-InS*. This excellent SPV response was attributed to not only activated T4-FeInS clusters dominating the migration of photogenerated holes onto the top electrode, but also to charge transfer from T3-InS clusters to T4-FeInS clusters, which prevented the instantaneous recombination of photogenerated charge carriers (Fig. S17†).

**Fig. 9:**
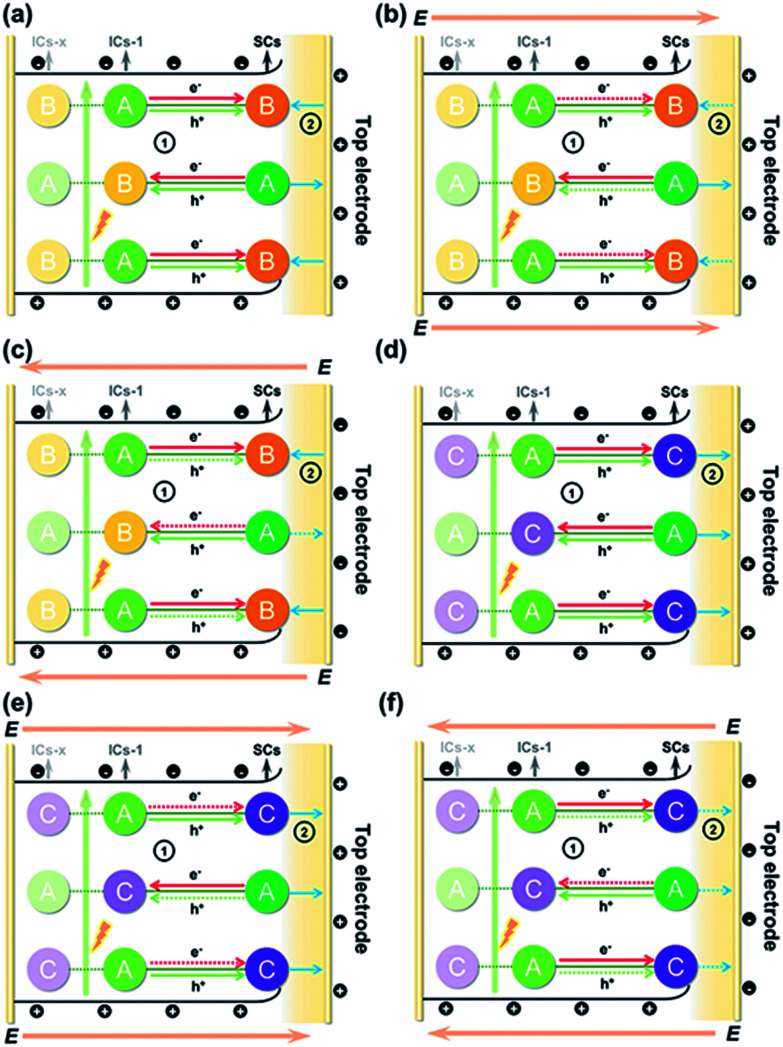
Schematic diagrams of photogenerated charge carrier migration under biases of (a and d) 0 V, (b and e) 10 V, and (c and f) −10 V for (a–c) *T3-T4-MnInS* and (d–f) *T3-T4-FeInS*. Note: A = T3-InS cluster; B = T4-MnInS cluster; C = T4-FeInS cluster; E = external electric field. ➀ refers to charge transfer between adjacent clusters (solid-line arrows: permitted or encouraged transfer; dotted-line arrows: unprocurable or suppressed transfer), while ➁ refers to the direction of the surface local electric field (solid-line arrows: consistent with the external electric field; dotted-line arrows: opposite to the external electric field).

The above results allowed us to rationally infer the dynamics of photogenerated charge carrier transfer between adjacent heteroclusters as well as between surface clusters and the top electrode in *T3-T4-MInS*. The accumulated charges on the top electrode were mainly derived from the photogenerated charges of surface clusters (SCs) and the charges migrated between SCs and interior adjacent clusters (ICs-1), which collectively relied on the external electric field, Schottky potential barrier, and staggered band gap alignment of *T3-T4-MInS*. The photogenerated charge could be spontaneously transferred from T3-InS clusters to adjacent T4-MInS clusters because of the encapsulated band gap alignment (Fig. 9a and d), which suppressed the instantaneous recombination of electron–hole pairs in the T3-InS cluster and thus prolonged the carrier lifetime of this T3-InS cluster and made the T4-MInS cluster charge-rich.

For *T3-T4-FeInS*, both T3-InS and T4-FeInS clusters dominated the transfer of holes to the top electrode because of the identical surface electric field (Fig. 9d). The synergistic effect of charge transfer within the heterocluster and the same Schottky potential barrier led to a much stronger SPV response of *T3-T4-FeInS* compared to that of *T3-InS* under a zero-electric field (Fig. 9f). However, *T3-T4-MnInS* featured a SPV performance dramatically different from that of *T3-T4-FeInS* and only exhibited a weak positive SPV signal due to the opposite local surface electric field direction in T3-InS and T4-MnInS regions (Fig. 8f and 9a). When a positive bias was applied to *T3-T4-MInS*, both electron transfer from T3-InS SCs to T4-MInS ICs-1 and hole transfer from T3-InS ICs-1 to T4-MInS SCs were promoted (Fig. 9b and e), while electron transfer from T3-InS ICs-1 to T4-MInS SCs and hole transfer from T3-InS SCs to T4-MInS ICs-1 were suppressed. Inversely, when a negative bias was applied, the dynamics of charge transfer between SCs and ICs-1 was opposite to that under a positive electric field, *i.e.*, electron transfer from T3-InS SCs to T4-MInS ICs-1 and hole transfer from T3-InS ICs-1 to T4-MInS SCs were suppressed, while electron transfer from T3-InS ICs-1 to T4-MInS SCs and hole transfer from T3-InS SCs to T4-MInS ICs-1 were promoted (Fig. 9c and f). As a result, all SCs including T3-InS and T4-MInS clusters in *T3-T4-MInS* were hole-rich under a positive electric field and electron-rich under a negative electric field. Charge migration from SCs to the top electrode was controlled by the external electric field and the additional surface electric field derived from the Schottky potential barrier. For *T3-T4-MnInS*, the external electric field was in the same direction as the surface electric field in the T3-InS regions of SCs but opposed the surface electric field in the T4-MnInS regions of SCs when a positive bias was applied. When a negative bias was applied, the external electric field was oriented in the same direction as the surface electric field in the T4-MnInS regions of SCs but opposed the surface electric field in the T3-InS regions of SCs (Fig. 9b and c). So, *T3-T4-MnInS* exhibited an obvious SPV response at both positive and negative biases, featuring a positive SPV signal weaker than that of *T3-InS* and a negative SPV signal stronger than that of *T4-MnInS*, which was ascribed to the photo-induced spontaneous charge transfer from T3-InS clusters to T4-MnInS clusters (Fig. 8f). For *T3-T4-FeInS*, both T3-InS SCs and T4-FeInS SCs dominated the transport of photogenerated holes to the electrode because of the Schottky potential barrier. Therefore, *T3-T4-FeInS* exhibited a preeminent photovoltaic response under positive bias due to the synergistic effect of the external electric field and the surface electric field as well as to charge transfer from T3-InS clusters to T4-FeInS clusters (Fig. 9e and f). It is worth noting that charge transfer processes between heteroclusters dramatically increased the utilization of photogenerated charges of T3-InS clusters, especially under an external electric field. Charge carriers transferred from T3-InS clusters to T4-MInS clusters cannot be devitalized *via* recombination, as redundant charges originating from the T4-MInS clusters are unavailable. For *T3-InS* and *T4-MnInS*, in the absence of applied bias, the charges of the top electrode mainly originated from SCs because of the absence of band gap alignment between adjacent T3-InS (or T4-MnInS) clusters, which allowed photogenerated charges to be spontaneously transferred from ICs-1 to SCs (Fig. S18a and d†). When an external bias was applied, charge transfer between SCs and ICs-1 was compulsively driven by the external electric field (Fig. S18b, c, e and f†). As a result, the SPV signal of *T3-InS* was weaker than that of *T3-T4-FeInS* when measured under a positive or negative electric field, and the signal of *T4-MnInS* was weaker than that of *T3-T4-MnInS* when measured under a negative electric field.

### Ultrafast transient absorption (TA) experiments

To further verify the dynamics of charge transfer from T3-InS clusters to T4-MInS clusters, we carried out nanosecond TA measurements on powdered samples of *T3-T4-MInS*, *T3-InS*, and *T4-MInS* using a pump pulse (355 nm)/white light continuum probe system (Fig. S19†). No TA signals were observed for *T3-InS*. This behavior was attributed to the instantaneous recombination of carriers and their relaxation from the exited S_*n*_ state to the S_1_ state within several nanoseconds or even picoseconds, which cannot be detected by a nanosecond laser (Fig. S20†). However, the other four samples exhibited distinct TA signals due to the existence of long-lifetime processes. As shown in Fig. S21,†*T4-MInS* showed focused TA bands at 500–650 nm (in *T4-MnInS*) and 395–450/640–700 nm (in *T4-FeInS*), while *T3-T4-MInS* showed obvious signals within the wide range of 350–700 nm. In addition, *T4-FeInS* exhibited strong ground-state bleaching at ∼380 nm, corresponding to its large extinction coefficient at 3 eV in steady-state absorption spectra. Long-lifetime processes in *T4-MInS* were attributed to charge transfer from the T4-MInS host to M^2+^ ions at the cluster center, followed by radiative or non-radiative recombination of electron–hole pairs in these metal ions. Importantly, the additional consecutive absorption band of *T3-T4-MInS* indicated the existence of other complicated absorbing states derived from charge transfer between T3-InS and T4-MInS clusters.^56^

**Fig. 10:**
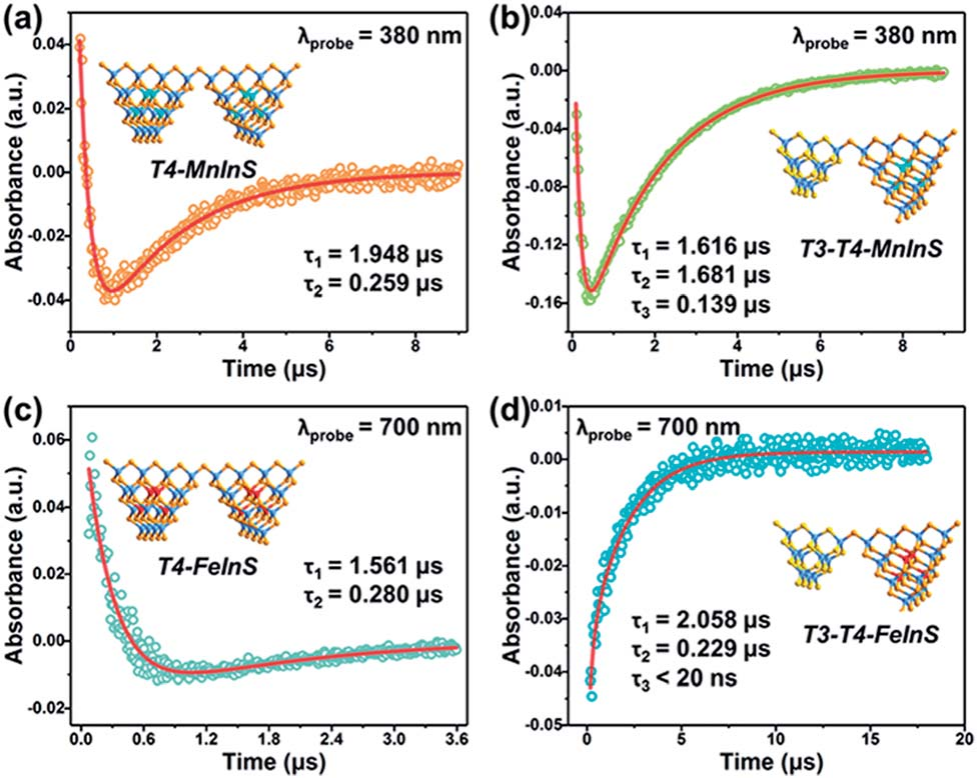
Representative TA kinetics of (a) *T4-MnInS* and (b) *T3-T4-MnInS* probed at 380 nm, and of (c) *T4-FeInS* and (d) *T3-T4-FeInS* probed at 700 nm. Insets show the sample structure.

Fig. 10 shows the TA dynamical curves of *T4-MInS* and *T3-T4-MInS* recorded for the probe wavelengths of 380 and 700 nm, and simulated *via* a global fit method to determine carrier lifetimes for all steps. The TA kinetic curves of the two *T4-MInS* were well fitted by bi-exponential functions (Fig. 10a and c), while those of the two *T3-T4-MInS* were well fitted by tri-exponential functions (Fig. 10b and d), *i.e.*, two decay processes operated in *T4-MInS* and three processes operated in *T3-T4-MInS* (Table S2†). For *T4-MInS*, the two fitted lifetimes were attributed to charge transfer from the host cluster to M^2+^ ions (longer decay time) and carrier recombination within M^2+^ dopants (shorter decay time). For *T3-T4-MInS*, in addition to these two processes, another short-lifetime decay process was ascribed to charge transfer from T3-InS clusters to adjacent T4-MInS clusters. Moreover, the lifetime of the second decay process (corresponding to the exciton transition of Mn^2+^ from the excited state to the ground state) in *T3-T4-MnInS* was almost seven-fold larger than that observed for *T4-MnInS*, which was consistent with the results of PL dynamics obtained by nanosecond transient emission spectroscopy (Fig. S22†).

## Conclusions

The dynamics of charge transfer in sub-nanoscale inorganic molecular heterojunctions was probed by deliberate selection of a group of crystalline heterocluster-based metal chalcogenides (*T3-T4-MInS*, M = Mn or Fe) as special structure models. Structural (size and component) differences between T3-InS and T4-MInS clusters resulted in separated band alignment, which enabled the transfer of photogenerated charge carriers between adjacent heteroclusters driven by different band potentials, as suggested by theoretical calculations. The double band edges in the UV-vis absorption spectra of *T3-T4-MInS* as well as the double PL emissions (T3-em and T4-em) of *T3-T4-MnInS* and the double SPV phase bands of *T3-T4-MInS* manifested the relative independence of T3-InS and T4-MInS clusters in the cluster-based frameworks, despite the existence of electron delocalization between them. The photogenerated charge carrier transfer from T3-InS clusters to T4-MInS clusters was further revealed by kinetics-dominated Mn^2+^-related PL intensity variation at different temperatures, long PL decay lifetimes of *T3-T4-MnInS*, and the quenching of T3-em PL in *T3-T4-FeInS*, and then proved by transient absorption spectroscopy. In addition, the SPV spectra of *T3-T4-MInS* measured at biases of 10 and −10 V demonstrated that charge carrier transfer between surface clusters and interior adjacent clusters obviously influenced charge aggregation at the top electrode. Thus, this pioneering research on the synergism of molecular building blocks, energy levels and dopants in controlling carrier dynamics provides a basis for the fabrication of next-generation photoelectric materials.

## Conflicts of interest

There are no conflicts to declare.

## Supplementary Material

SC-011-D0SC00458H-s001

SC-011-D0SC00458H-s002
