# Supplementary material for: Direct observation of charge transfer between molecular heterojunctions based on inorganic semiconductor clusters
Source: Chem Sci. 2020 Mar 23;11(16):4085–96. doi: 10.1039/d0sc00458h (PMC8152627; doi:10.1039/d0sc00458h)
Supplement: SC-011-D0SC00458H-s001 [file SC-011-D0SC00458H-s001.pdf]

Electronic Supplementary Material (ESI) for Chemical Science.
This journal is © The Royal Society of Chemistry 2020

Supporting Information for

Direct Observation of Charge Transfer between Molecular Heterojunctions Based on Inorganic Semiconductor Clusters

Chaozhuang Xue,[‡a] Xing Fan,[‡b] Jiaxu Zhang,[a] Dandan Hu,[a] Xiao-Li Wang,[a] Xiang Wang,[a] Rui Zhou,[a] Haiping Lin,[b] Youyong Li,[b] Dong-Sheng Li,[c] Xiao Wei,[d] Daoyuan Zheng,[e,f] Yang Yang,[e] Keli Han,[e] and Tao Wu*[a]

[a] College of Chemistry, Chemical Engineering and Materials Science, Soochow University, Jiangsu 215123, China.

[b] Institute of Functional Nano & Soft Materials (FUNSOM), Jiangsu Key Laboratory for Carbon-Based Functional Materials & Devices, Soochow University, Suzhou, Jiangsu 215123, China.

[c] College of Materials and Chemical Engineering, Hubei Provincial Collaborative Innovation Center for New Energy Microgrid, Key Laboratory of Inorganic Nonmetallic Crystalline and Energy Conversion Materials, China Three Gorges University, Yichang 443002, China.

[d] School of Chemistry and Chemical Engineering, Shanghai Jiao Tong University, Shanghai 200240, China.

[e] State Key Laboratory of Molecular Reaction Dynamics, Dalian Institute of Chemical Physics, Chinese Academy of Science, Dalian 116023, China.

[f] Institute of Molecular Sciences and Engineering, Shandong University, Qingdao 266235, China.

Corresponding Author: wutao@suda.edu.cn

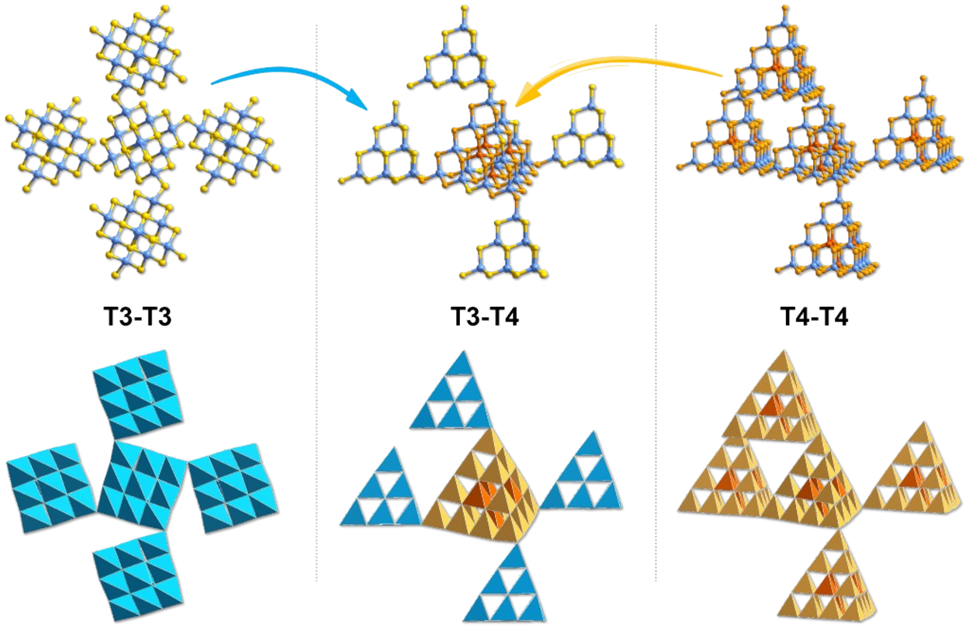

Fig. S1 Structure illustration of tetrahedrally-connected clusters: T3-T3 in *T3-InS* (left), T3-T4 in *T3-T4-MInS* (middle) and T4-T4 in *T4-MInS* (right).

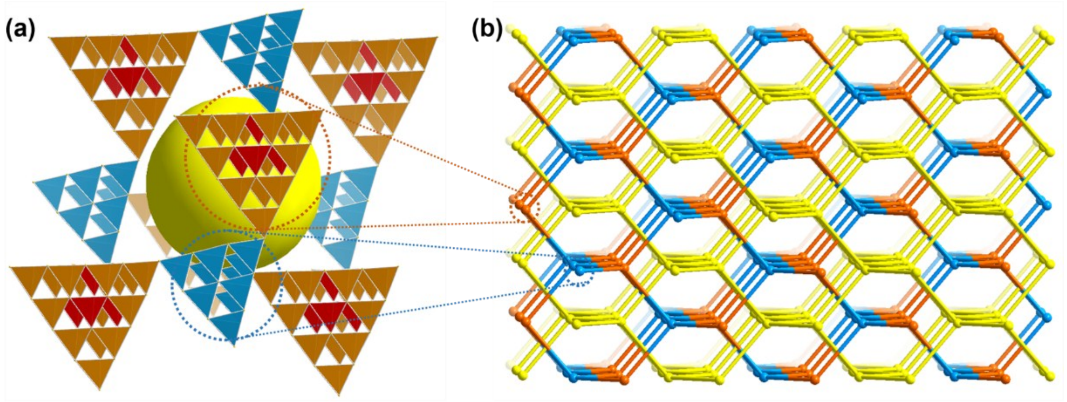

Fig. S2 (a) An adamantane cage constructed by interlinked T3-InS clusters and T4-MInS clusters (M = Mn or Fe). (b) The double-interpenetrated *dia* network of *T3-T4-MInS* when each of T3-InS and T4-MInS cluster is treated as four-connected node.

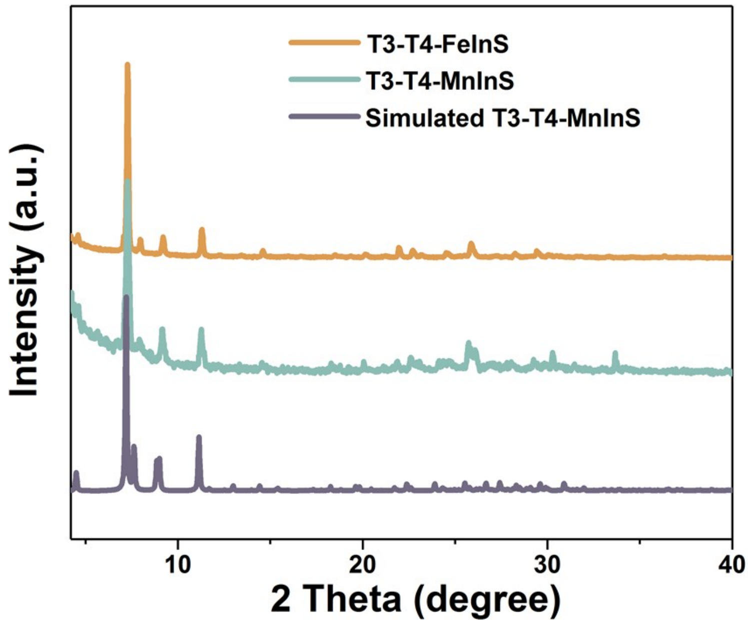

Fig. S3 PXRD patterns of *T3-T4-MnInS* and *T3-T4-FeInS*.

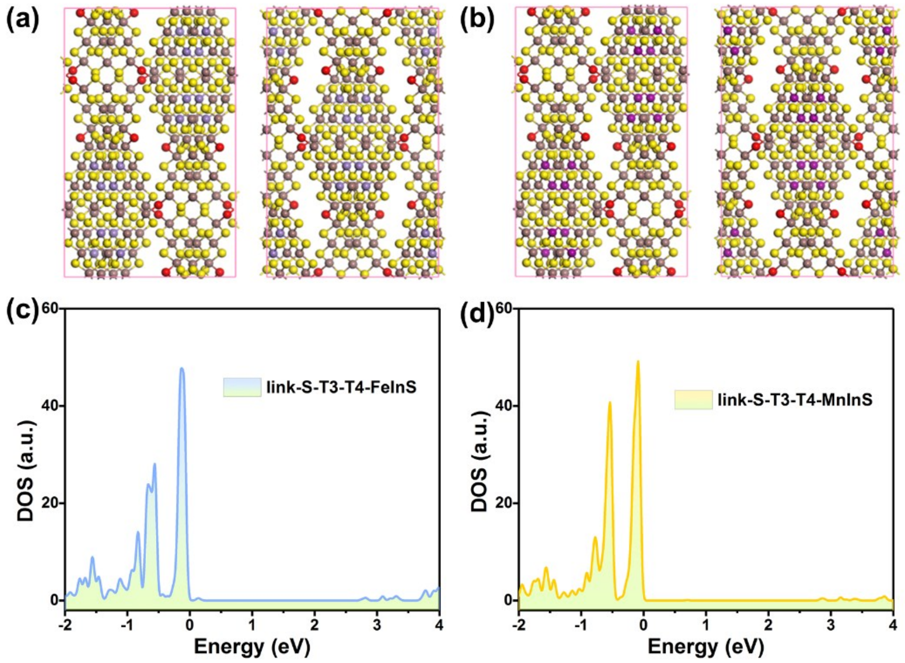

Fig. S4 The atomic positions of linker S atoms in the (a) *T3-T4-FeInS* and (b) *T3-T4-MnInS.* The PDOS plots of linker S atoms in the (c) *T3-T4-FeInS* and (d) *T3-T4-MnInS*. Color code: In is brown, Fe is blue, Mn is violet, linker S is red, other S is yellow.

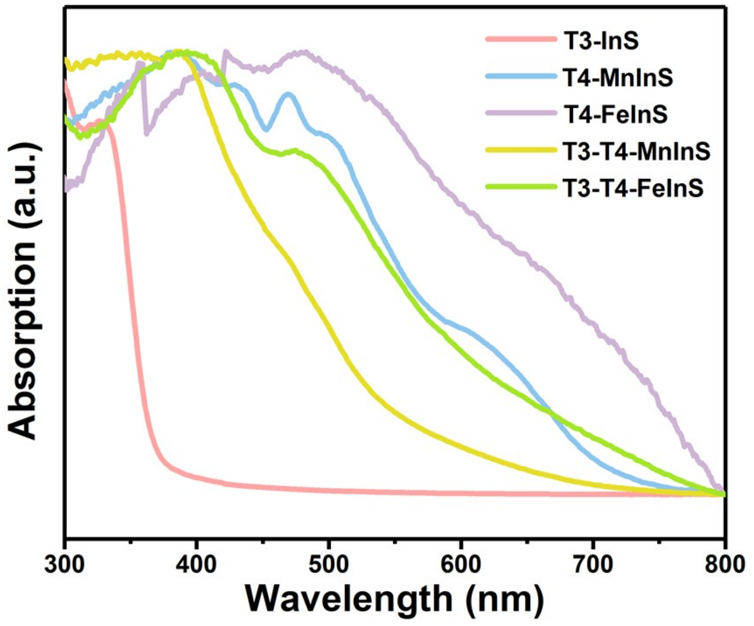

Fig. S5 UV-vis absorption spectra of pure *T3-InS*, *T4-MInS*, and hybrid *T3-T4-MInS*.

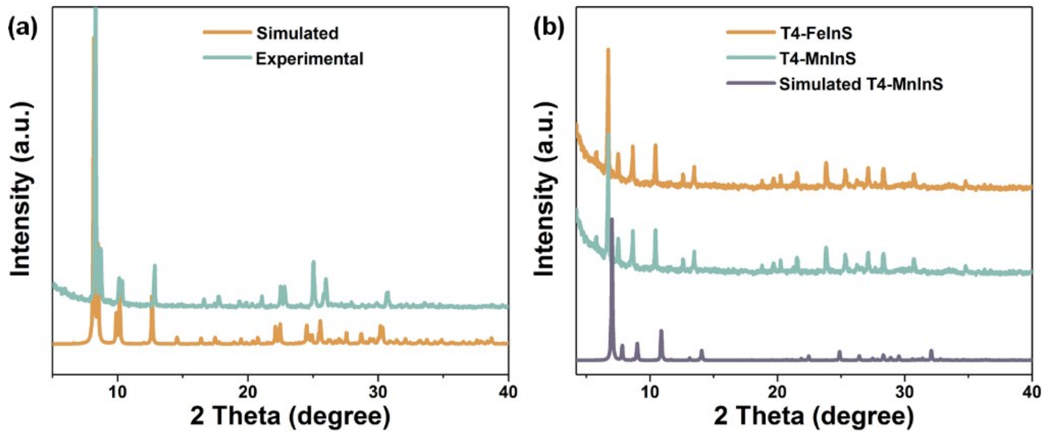

Fig. S6 PXRD patterns of (a) pure *T3-InS* and (b) pure *T4-MInS*.

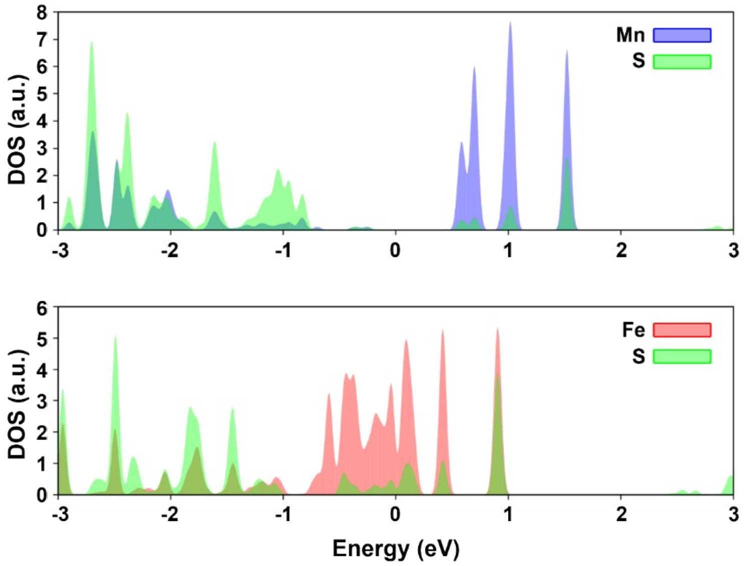

Fig. S7 The PDOS plots of (a) the bonded Fe and S atoms in T4-FeInS cluster; (b) the bonded Mn and S atoms in T4-MnInS cluster. It is seen that hybridization of Fe atoms are much more profound than that of Mn atoms.

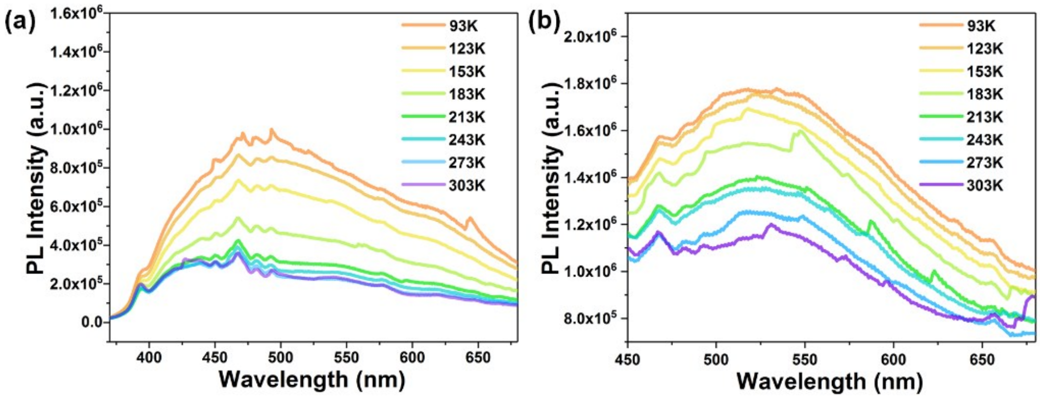

Fig. S8 PL spectra of pure *T3-InS* under excitation of (a) 350 nm and (b) 400 nm.

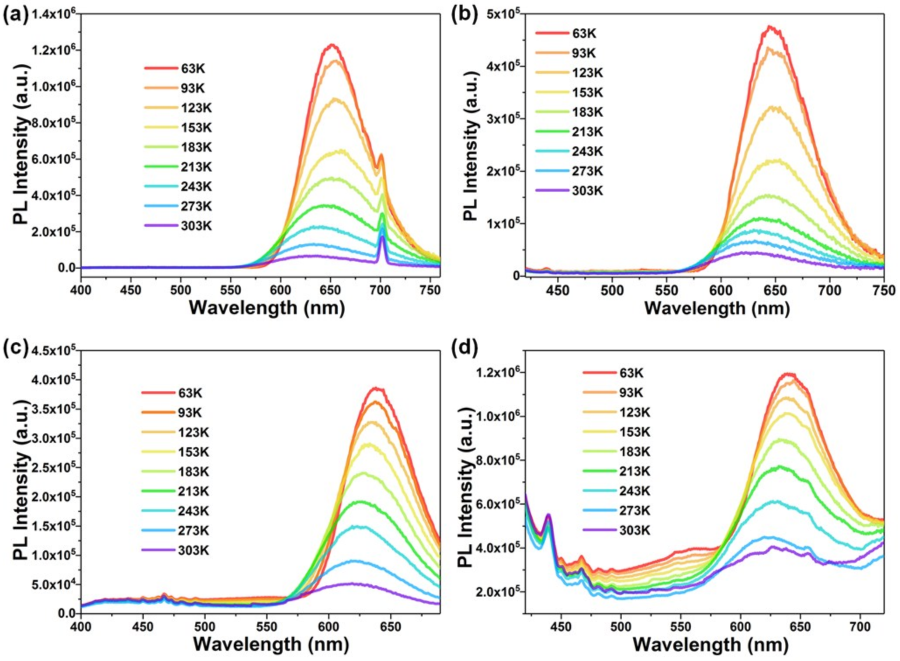

Fig. S9 PL spectra of pure *T4-MnInS* under excitation of (a) 350 nm and (b) 400 nm. PL spectra of hybrid *T3-T4-MnInS* under excitation of (c) 350 nm and (d) 400 nm.

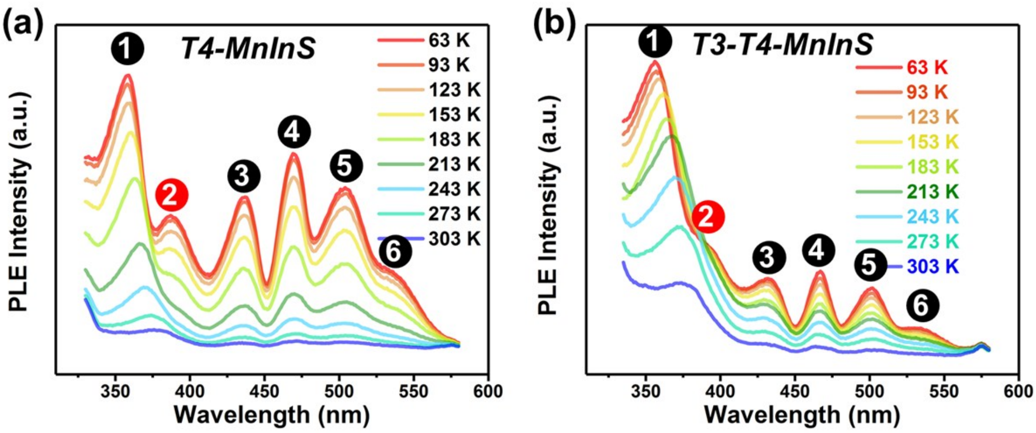

Fig. S10 Temperature-dependent PLE spectra of (a) *T4-MnInS* and (b) *T3-T4-MnInS* at the emission wavelength of 631 nm. **Note:** 1: energy transfer path from the host to Mn^{2+} ions; 2: $^6A_1(^6S)\rightarrow{}^4E(^4D)$; 3: $^6A_1$ $(^6S)\rightarrow{}^4T_2(^4D)$; 4: $^6A_1(^6S)\rightarrow{}^4E(^4G)$ and $^4A_1(^4G)$; 5: $^6A_1(^6S)\rightarrow{}^4T_2(^4G)$; 6: $^6A_1(^6S)\rightarrow{}^4T_1$ (^4G).

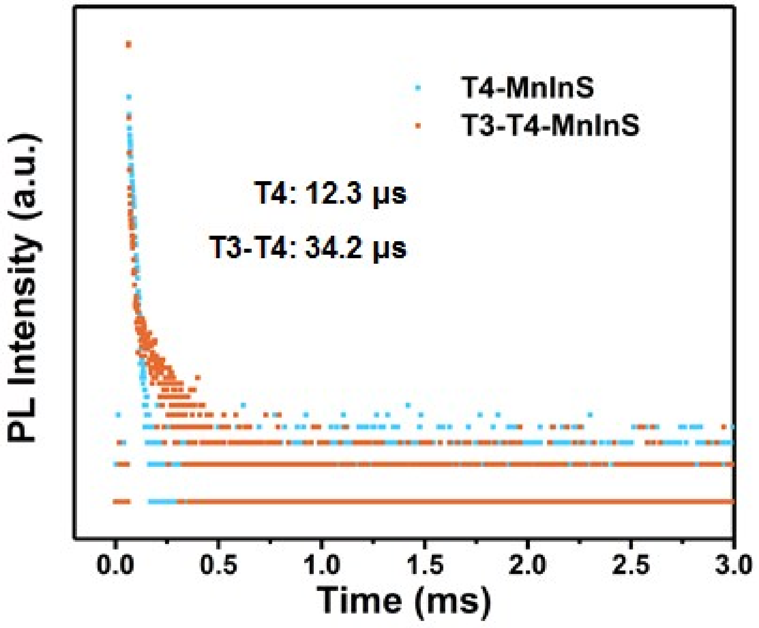

Fig. S11 Room-temperature PL decay curve of hybrid *T3-T4-MnInS* and pure *T4-MnInS* under excitation at 504 nm excitation and detected at emission of 631 nm.

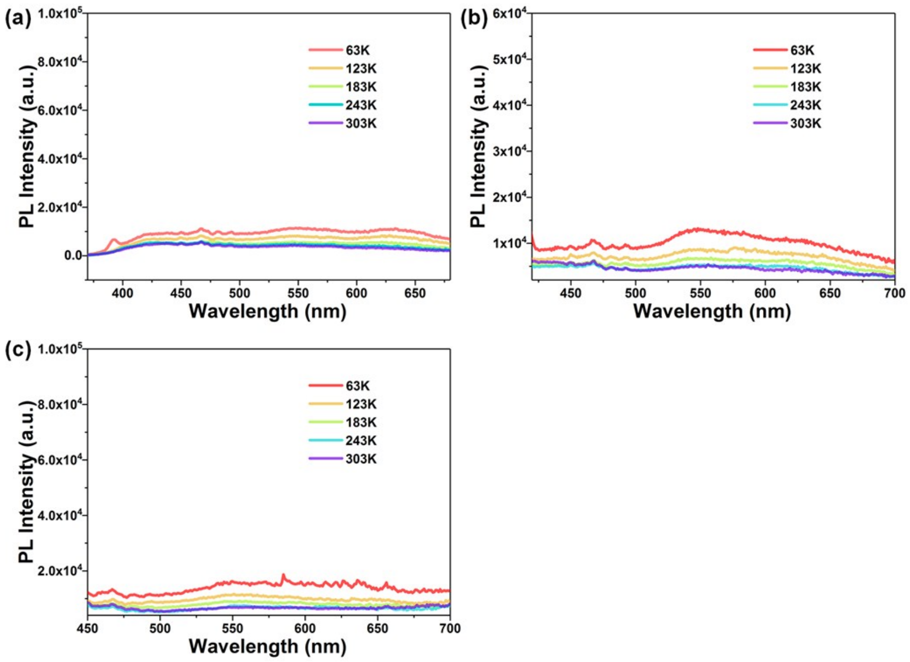

Fig. S12 PL spectra of pure *T4-FeInS* under excitation of (a) 350 nm, (b) 375 nm and (c) 400 nm.

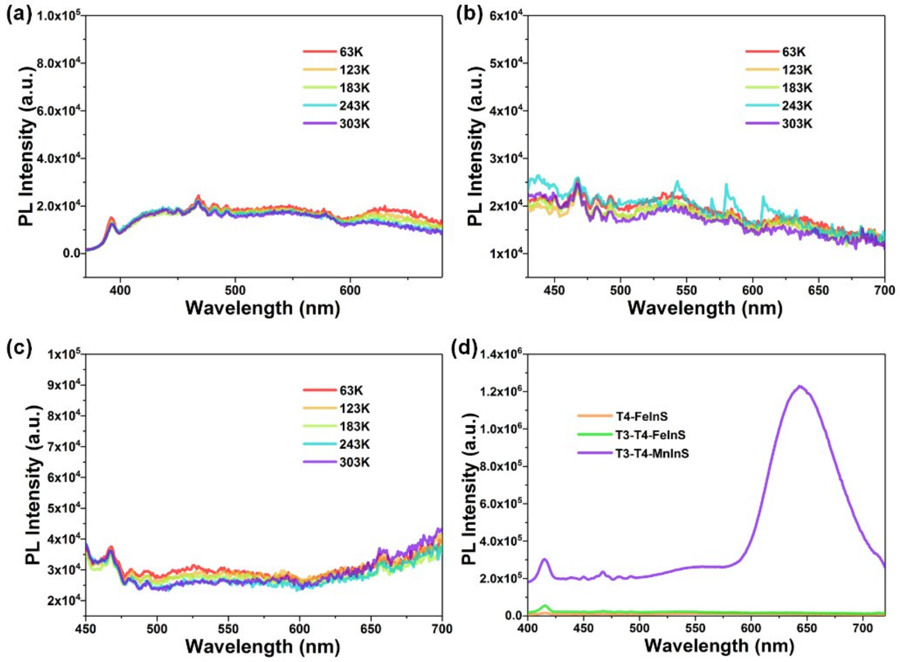

Fig. S13 PL spectra of hybrid *T3-T4-FeInS* under excitation of (a) 350 nm, (b) 375 nm and (c) 400 nm. (d) PL emission of pure *T4-FeInS*, hybrid *T3-T4-FeInS* and hybrid *T3-T4-MnInS*.

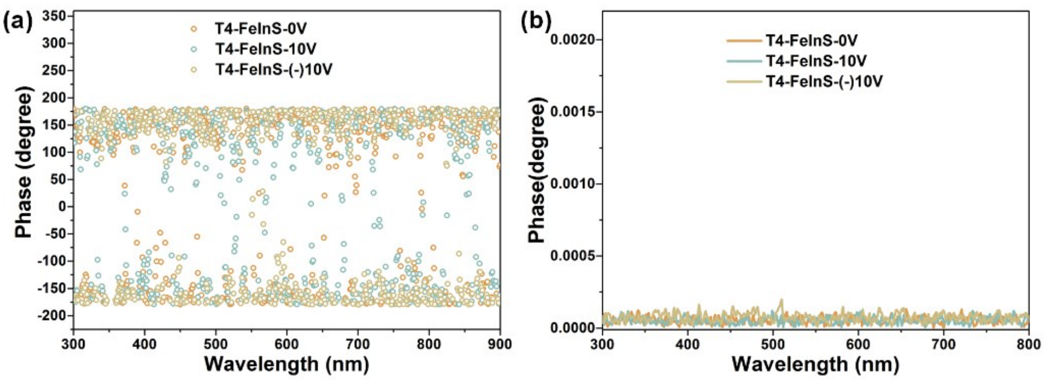

Fig. S14 (a) SPV phase spectra and (b) SPV spectra of pure *T4-FeInS* under biases of 0 V, 10 V and -10 V.

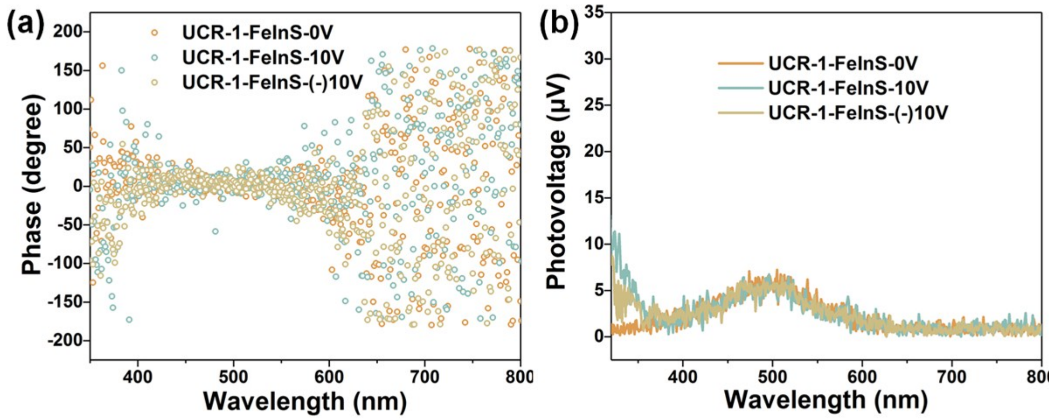

Fig. S15 (a) SPV phase spectra and (b) SPV spectra of *UCR-1-FeInS* under biases of 0 V, 10 V and -10 V.

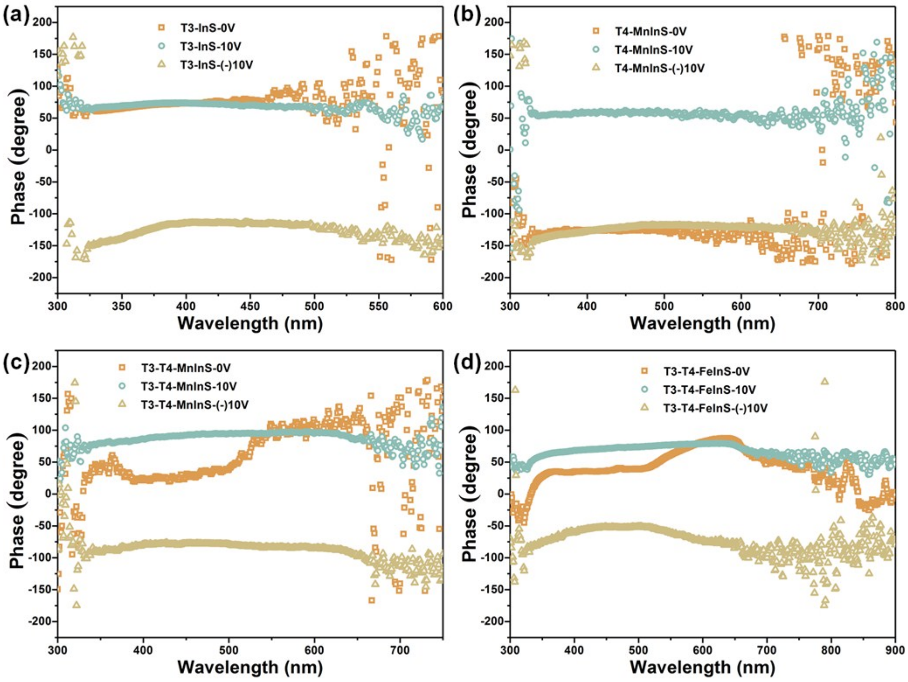

Fig. S16 SPV phase spectra of (a) pure *T3-InS*, (b) pure *T4-MnInS*, and (c) hybrid *T3-T4-MnInS*, (d) hybrid *T3-T4-FeInS* under biases of 0 V, 10 V and -10 V.

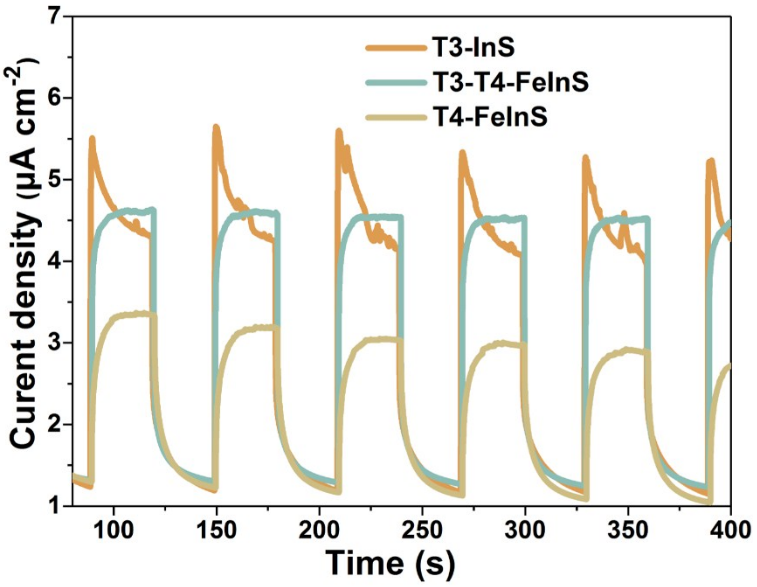

Fig. S17 Photoelectric response curves versus time (*J–t*) of pure *T3-InS*, *T4-FeInS* and hybrid *T3-T4-FeInS* measured at 0.6 V vs. Hg/Hg_2Cl_2 in 0.3 M Na_2SO_4 electrolyte under irradiation of 300 W high pressure xenon lamp.

To prepare the electrode for photoelectrochemical measurements, 2 mg of the sample were first ground into fine powders using a marble and pestle, and then added into 15 mL isopropanol. The mixture was then subjected to sonication in a water bath until the sample was homogeneously dispersed. The ITO slices were sonicated in acetone and water, respectively, for about 15 min. After that, 100 μL of the compound suspensions were dropped onto the dried surface of ITO substrate with about surface area of 1.0 cm^2 and then dried at room temperature for photocurrent measurements. The photocurrent measurements were performed on a CHI 670D electrochemistry potentiostat in a standard three-electrode configuration with the sample-coated ITO as the working electrode, the Hg/Hg_2Cl_2 as the reference electrode, and the Pt foil as the counter electrode. A full-spectrum illumination was provided by a 300 W Xe lamp (20 cm away from working electrode). 0.3 M Na_2SO_4 solution purged with N_2 for 30 min before measurement was used as electrolyte. The linear sweep voltammetry was conducted from -0.2 to 0.6 V (*vs.* Hg/Hg_2Cl_2) with a scan rate of 10 mV/s. The photoelectrochemical response curves were obtained at 0.6 V (*vs.* Hg/Hg_2Cl_2) under chopped illumination.

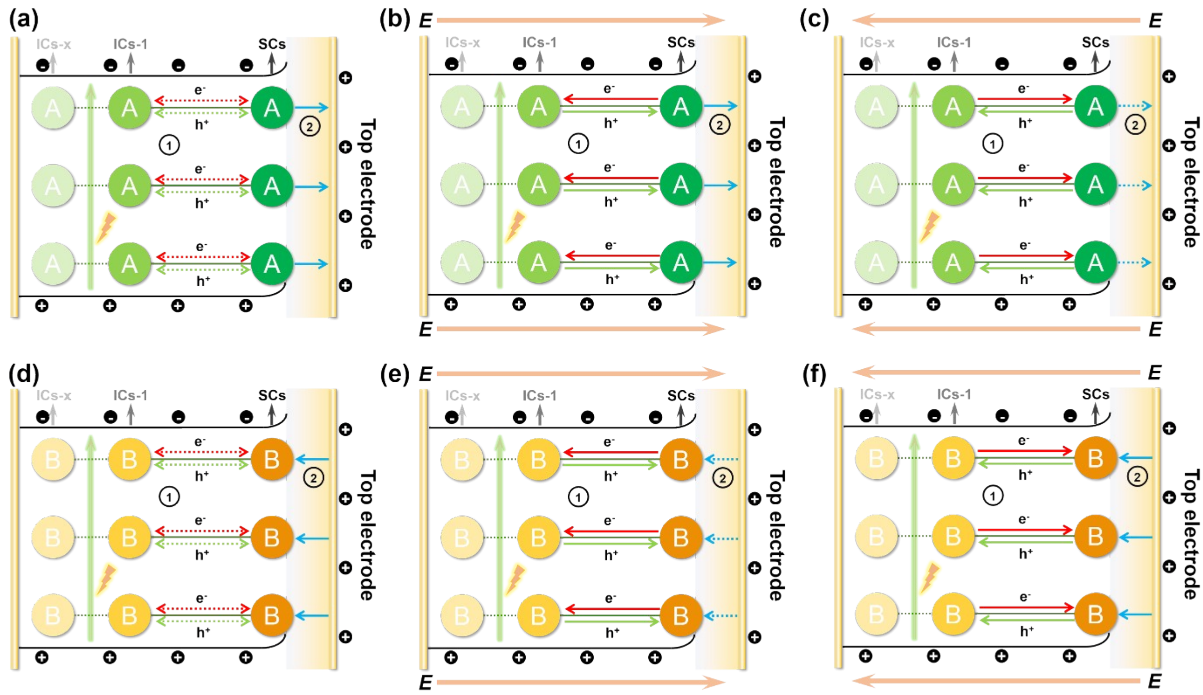

Fig. S18 Schematic diagrams of the photogenerated carriers' migration: under bias of 0 V (a), 10 V (b) and -10 V (c) for pure T3-InS; under bias of 0 V (d), 10 V (e) and -10 V (f) for pure T4-MnInS; E, external electric field. The number ① refers to charge transfer process between adjacent clusters (arrows with solid line: permitted or encouraged transfer process; arrows with dot line: unprocurable or suppressed transfer process). The number ② refers to surface local electric field direction (arrows with solid line: consistent with external electric field; arrows with dot line: opposite to external electric field).

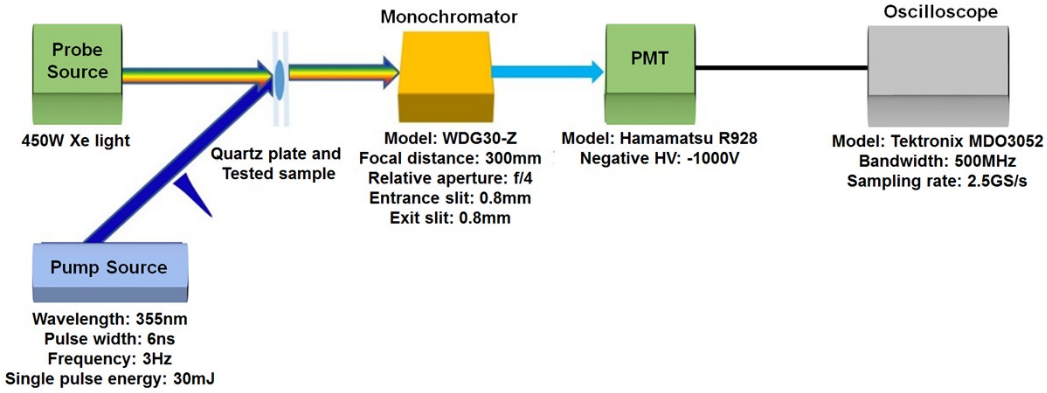

Fig. S19 Schematic diagram of system for ultrafast transient absorption (TA) and emission measurements.

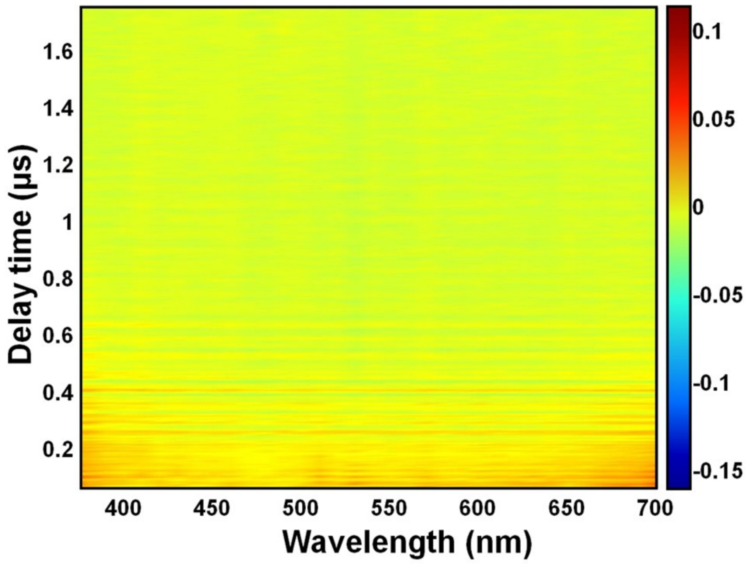

Fig. S20 Transient absorption map of pure *T3-InS* under excitation of 355 nm.

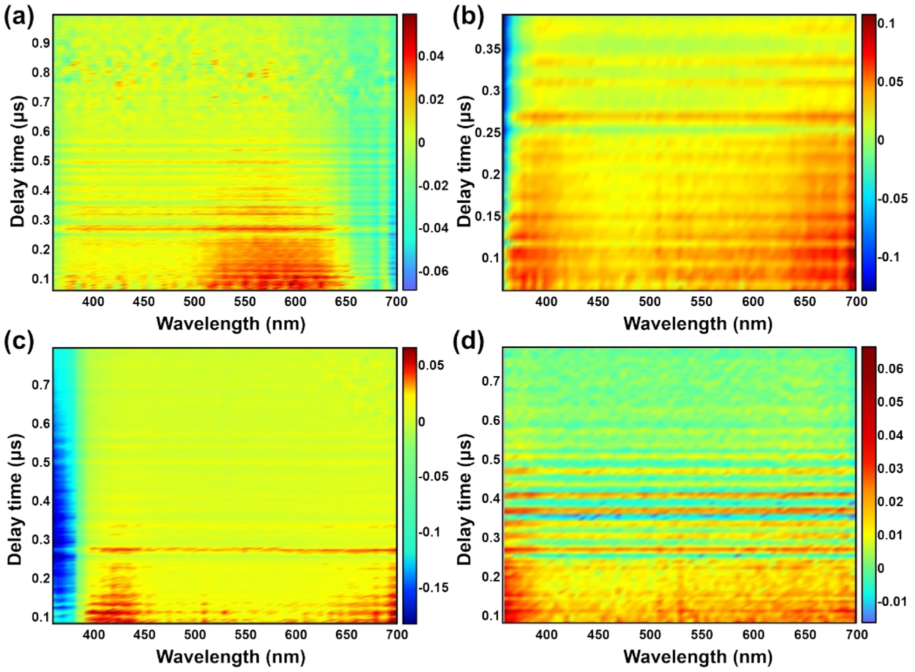

Fig. S21 Transient absorption map of (a) pure *T4-MnInS*, (b) hybrid *T3-T4-MnInS*, (c) pure *T4-FeInS* and (d) hybrid *T3-T4-FeInS* under excitation of 355 nm.

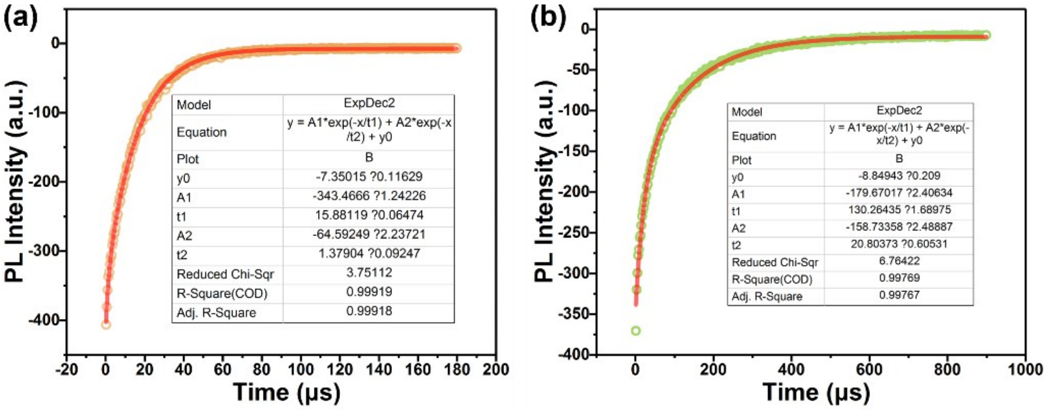

Fig. S22 PL decay curves of (a) pure *T4-MnInS* and (b) hybrid *T3-T4-MnInS* obtained by nanosecond transient emission measurements and fitted with exponential method. The PL lifetime of T4-MnInS and T3-T4-MnInS were calculated to be about 15.65 μs and 116.77 μs, respectively.

The discrepancy of PL lifetimes occurring in Figure 6 and Figure S22 can be explained as following. In order to obtain rigorous and meticulous experiments, we utilize two different types of instruments for cross validation of our conclusion. The first instrument name by HORIBA Scientific Fluorolog-3 is based on time-correlated single photon counting (TCSPC) technology, which is used to detect single photon, while the second instrument named by Nanosecond Transient Absorption and Emission Spectroscopy (NTAS) is based on direct photo-electric conversion, which is used to detect relatively strong light beam or high photon flux. NTAS has two working modes. One mode is to measure transient emission spectra, and the other mode is to measure transient absorption spectra. Without any deconvolution process, the time resolutions of HORIBA Scientific Fluorolog-3 and NTAS are within 2 ns and 20 ns, respectively, both of which are high enough for our samples. Therefore, we think that the curves in Figure 6 in the Text and Figure S22 in the Supporting Information file are both correct. Due to the different working principles in HORIBA Scientific Fluorolog-3 and NTAS, the intensities of excitation pulse light are quite different, which cause the disparity in the measured lifetimes from two different instruments. Therefore, we have a better understanding of the disparity in the measured lifetimes from two different instruments. On one hand, the excitation light intensity in HORIBA Scientific Fluorolog-3 cannot be measured by the energy meter, which means its energy per pulse should be less than 0.1 mJ. In standard TCSPC, due to the weak excitation, each pulse can only excite a small number of ground-states, resulting in the generation of very few electron-hole separation states. On the other hand, in NTAS measurement, the

excitation light with 8 mm beam diameter is 10 mJ@355mn without being focused. Obviously, the excitation intensity in NTAS is at least 2 orders higher than that in HORIBA Scientific Fluorolog-3. The intense excitation in NTAS not only helps with getting high signal-to-noise data, as shown in Figure S22, but also generates a large number of electron-hole separation states. Then the strong coulomb interactions between electrons or holes accelerate the recombination process, resulting in the shorter lifetime. Hence, it is much reasonable that the lifetime obtained by NTAS is much shorter.

Table S1. PL Lifetime of pure *T4-MnInS*, hybrid *T3-T4-MnInS* and the mechanically mixed *T3-InS* and *T4-MnInS* monitored at the emission wavelength of 631 nm.

**Samples**	λ_{Ex}	**Parameter (%)**	**Value (τ in *s*)**	R^2
**T4-MnInS**	372	A1	6.72	**1.001762**
		τ1	2.193038E-05	
		A2	36.67	
		τ2	4.3179E-05	
		A3	56.61	
		τ3	7.015749E-05	
**T3-T4-MnInS**		A1	39.04	**0.969366**
		τ1	1.797632E-04	
		A2	52.96	
		τ2	3.670506E-04	
		A3	8.00	
		τ3	4.862797E-06	
**T3-InS + T4-MnInS**		A1	1.72	**1.063104**
		τ1	7.095042E-07	
		A2	61.20	
		τ2	2.954797E-05	
		A3	37.08	
		τ3	5.93972E-05	
**T4-MnInS**	469	A1	8.64	**0.925651**
		τ1	1.334059E-06	
		A2	88.70	
		τ2	1.501769E-07	
		A3	2.66	
		τ3	2.37082E-07	
**T3-T4-MnInS**		A1	40.13	**1.003243**
		τ1	1.1005E-05	
		A2	49.76	
		τ2	9.32408E-05	
		A3	10.11	
		τ3	1.767628E-05	

Table S2. The exponential fitted parameters of transient absorption dynamical curves of pure *T4-MInS* and hybrid *T3-T4-MInS*.

**Samples**	**Wavelength (nm)**	**Parameter**	**Value (τ in μs)**	**Standard Error**	R^2
**T4-MnInS**	380	y0	9.825E-5	2.839E-4	**0.96774**
		A1	0.2351	0.00577	
		τ1	0.25892	0.008	
		A2	-0.07058	0.00185	
		τ2	1.94774	0.061	
**T3-T4-MnInS**		y0	-1.6615E-4	0.0012	**0.99735**
		A1	-0.75061	458.2334	
		τ1	1.68139	0.38263	
		A2	0.54082	458.23095	
		τ2	1.61578	27.3298	
		A3	0.3387	0.00552	
		τ3	0.13939	0.00412	
**T4-FeInS**	700	y0	3.7889E-4	0.00158	**0.94378**
		A1	-0.02358	0.00312	
		τ1	1.56085	0.45253	
		A2	0.09619	0.00366	
		τ2	0.27992	0.01633	
**T3-T4-FeInS**		y0	0.0014	1.03172E-4	**0.9637**
		A1	0.01752	6.14254E11	
		τ1	0.00695	9.40801E9	
		A2	-0.03792	0.00131	
		τ2	2.05798	0.06932	
		A3	-0.02106	0.01059	
		τ3	0.22877	0.09215	
